# Diverse proteins aggregate in mild cognitive impairment and Alzheimer’s disease brain

**DOI:** 10.1186/s13195-020-00641-2

**Published:** 2020-06-19

**Authors:** Devin Kepchia, Ling Huang, Richard Dargusch, Robert A. Rissman, Maxim N. Shokhirev, Wolfgang Fischer, David Schubert

**Affiliations:** 1 Cellular Neurobiology Laboratory, The Salk Institute for Biological Studies, La Jolla, CA 92037 USA; 2 The Razavi Newman Integrative Genomics and Bioinformatics Core, The Salk Institute for Biological Studies, La Jolla, CA 92037 USA; 3 Department of Neurosciences and Shiley-Marcos Alzheimer’s Disease Research Center Neuropathology Core, University of California, San Diego, La Jolla, CA 92093 USA

**Keywords:** Dementia, Insoluble protein, Secondary modifications, Proteomics, Bioinformatics

## Abstract

**Background:**

All cells accumulate insoluble protein aggregates throughout their lifespan. While many studies have characterized the canonical disease-associated protein aggregates, such as those associated with amyloid plaques, additional, undefined proteins aggregate in the brain and may be directly associated with disease and lifespan.

**Methods:**

A proteomics approach was used to identify a large subset of insoluble proteins in the mild cognitively impaired (MCI) and Alzheimer’s disease (AD) human brain. Cortical samples from control, MCI, and AD patients were separated into detergent-soluble and detergent-insoluble fractions, and high-resolution LC/MS/MS technology was used to determine which proteins became more insoluble in the disease state. Bioinformatics analyses were used to determine if the alteration of protein aggregation between AD and control patients was associated with any specific biological process. Western blots were used to validate the proteomics data and to assess the levels of secondary protein modifications in MCI and AD.

**Results:**

There was a stage-dependent increase in detergent-insoluble proteins, with more extreme changes occurring in the AD cohort. Glycolysis was the most significantly overrepresented gene ontology biological process associated with the alteration of protein aggregation between AD and control patients. It was further shown that many low molecular weight proteins that were enriched in the AD brain were also highly aggregated, migrating on SDS-PAGE far above their predicted molecular masses. Glucose-6-phosphate isomerase, ubiquitin carboxyl-terminal hydrolase isoenzyme L1 (UCHL1/PARK5), and the DNA damage repair enzyme KU70 were among the top insoluble proteins identified by proteomics and validated by Western blot to be increased in the insoluble fractions of both MCI and AD brain samples.

**Conclusions:**

Diverse proteins became more detergent-insoluble in the brains of both MCI and AD patients compared to age-matched controls, suggesting that multiple proteins aggregate in these diseases, likely posing a direct toxic insult to neurons. Furthermore, detergent-insoluble proteins included those with important biological activities for critical cellular processes such as energetics, proteolysis, and DNA damage repair. Thus, reduced protein solubility likely promotes aggregation and limits functionality, reducing the efficiency of multiple aspects of cell physiology. Pharmaceutical interventions that increase autophagy may provide a useful therapeutic treatment to combat protein aggregation.

## Background

As cells age, they accumulate insoluble protein aggregates. This is observed in all species ranging from bacteria to human neurons and is elevated in a number of human diseases [1]. The aggregation of specific proteins, such as amyloid beta (Aβ), phosphorylated tau, and α-synuclein, is usually associated with the neurodegenerative diseases of aging. However, there is evidence that additional undefined, detergent-insoluble, aggregated proteins accumulate in the brain during the aging process and may be directly associated with disease and lifespan.

Previously, our laboratory identified a new class of detergent-insoluble proteins in flies whose levels dramatically increase in the brain with age [2]. When these proteins were removed by increasing autophagy specifically in the brain, the flies lived longer, while increasing aggregated proteins by decreasing autophagy in the brain decreased lifespan [2]. This experiment was later repeated in *C. elegans* with similar results [3]. Therefore, at least in flies and worms, the aggregation of proteins is directly linked to a decrease in longevity. Detergent-insoluble aggregates also accumulate with age in Alzheimer’s disease (AD) transgenic mice and are reduced by neuroprotective AD drug candidates that extend lifespan in worms and flies [4–6].

Previous gene expression and proteomics studies in the AD brain have largely focused upon global changes [7–10] and the oxidatively modified proteins [11]. In addition, using different criteria for solubility than those that were used here, protein aggregates were examined in a mouse AD model [12] and in human brain [13, 14]. Here we determined the identity of old age-associated insoluble proteins and their secondary modifications in both mild cognitively impaired (MCI) and AD cortices and characterized their potential associations with cell viability and the progression of AD-associated brain pathology.

To determine if brain tissue from AD patients recapitulates our observations in transgenic AD mice, we asked if there was an increase in specific detergent-insoluble proteins in the AD brain relative to age- and sex-matched controls. We also examined the levels of the same subset of proteins in MCI cortical tissue. Because aggregated proteins can be either soluble or insoluble in a detergent, and the fact that our assay involves proteins in high-speed centrifugation pellets, we call this set of proteins the pelletome. It is shown that there was a unique subset of proteins that were more abundantly expressed in the pelletome of the AD cortex compared to age- and sex-matched controls and that many of these proteins remained bound tightly to each other in the presence of a detergent. These proteins were identified and bioinformatics analyses determined that glycolysis was the most significantly overrepresented gene ontology (GO) biological process associated with the alteration of protein aggregation between AD and control patients. An analysis of secondary modifications by Western blotting showed that lysines were differentially modified between AD and control groups, suggesting a change in protein catabolism with the disease.

## Methods

### Profile of subjects used in this study

Postmortem fresh frozen cortical tissues were obtained from the University of California, San Diego (UCSD) Shiley-Marcos Alzheimer’s Disease Research Center (ADRC) Neuropathology Core. Autopsy-validated, de-identified tissues were obtained from Broadmann area 9 of the frontal cortex of eight age- and sex-matched (female) control patients and eight AD patients (Supplementary Table 1). The average age of both groups was 87 years. Control patients had no cognitive impairment with normal neuropsychological tests and daily living scores. Additionally, tissues were obtained from Broadmann area 9 of the frontal cortex of 10 MCI and 10 control patients from UCSD (Supplementary Table 2). The MCI samples were from both sexes and the average age of both groups was 78 years.

### Proteomics

Human cortical brain tissue (100 mg) was homogenized by sonication in RIPA buffer (1 ml, 50 mM Tris, pH 7.5, 150 mM NaCl, 1% NP-40, 0.1% SDS, 0.5% deoxycholate). Cellular debris was removed by low-speed centrifugation (5000×*g* for 5 min). This was followed by high-speed centrifugation (average RCF 81,000×*g* for 1 h). The pellet was washed once with RIPA buffer. For further processing, pellets were solubilized in 1 ml buffer containing 6 M urea, 2% SDS, 50 mM Tris, pH 7.5, and 50 mM DTT by sonication (20 s) and incubated at 60 °C for 15 min. For trypsin digestion, RIPA-soluble material and solubilized RIPA-insoluble material were processed by gel-aided sample preparation [15]. Digests were analyzed by high-resolution LC/MS/MS on a Thermo Orbitrap Fusion instrument. Raw mass spectral data were searched by employing an IP2 Integrated Proteomics Applications cluster. Relative quantitation was achieved by comparing spectral counts using the ID-Stat-Compare feature of the program.

### Bioinformatics

A pseudo count of 5 was added to the Raw Mass Spectral (MS) counts to avoid infinity during log2-transformation. Then the log counts were quantile-normalized to minimize sample technical variability. In order to account for individual specific protein expression patterns, the pellet-to-soluble protein ratio was calculated based upon the log fold difference between pellet and soluble fractions of the same protein per individual. A positive protein pellet-to-soluble ratio indicated preferential accumulation in the pellet fraction whereas a negative number indicated preferential accumulation in the soluble fraction. Principle component analysis (PCA) was performed on the centered pellet-to-soluble ratios using the R function prcomp. A two-tailed *t* test was used to test for the difference in the protein pellet-to-soluble ratios between AD brains and control brains. Proteins with adjusted *P* value < 0.05 and absolute logFC > 0.5 were identified as significantly differentially accumulated. DAVID Bioinformatics enrichment tool [16] was used to identify the most overrepresented gene ontology (GO) terms involved with AD-associated differentially accumulated proteins. All statistics and figures were generated using R statistical environment unless mentioned specifically [17]. R packages ggplot2 [18], VennDiagram [19], and gplots [20] were used for figure plotting.

### Western blotting

Equal amounts of protein were homogenized and solubilized in 2x SDS-sample buffer, separated on 4 to 12% SDS-polyacrylamide gels, transferred to Immobilon-P and immunoblotted. Gels and blots were scanned on a Bio-Rad ChemiDoc MP imaging system. Protein levels were normalized to total protein using Bio-Rad Stain-Free imaging technology. An unpaired two-tailed *t* test was performed to compare between two groups. All statistical analysis was conducted using Prism software. The raw Western blots are shown in the Supplementary Figures.

Unless otherwise indicated, antibodies were from Cell Signaling Technologies (CS) or AbCAM (Ab) and used at a 1 to 2000 dilution. The molecular weights of the proteins shown in the Western blots are also included along with the catalog numbers: Tau, CS4019, 50–80 kDa; Amyloid Beta, 6E10, Signet9320; ApoE (pan), CS13366, 35 kDa; PGAM, CS11896, 28 kDa; UCHL1, CS11896, 27 kDa; KU70, CS4588, 70 kDa; ADCY1, ABclonalA9760, 100 kDa; CAMK2 (pan), CS4436, 50 kDa; FASN, CS3189, 273 kDa; GPI, CS57893S, 60 kDa; CKB, Ab92452, 43 kDa; LRP, CS64099, 85 kDa; HSP90β, CS7411S, 90 kDa; HSP75, CS95345S, 75 kDa; HSP70, CS9965, 70 kDa; HSP40, CS9965, 40 kDa; Coronin, CS60615, 60 kDa; Drebrin, CS5052, 120 kDa; Tubulin, CS2148, 52 kDa; Gelsolin, CS12953, 83 kDa; Dynamin 1, CS4565, 100 kDa; 14–3-3, CS8312, 27 kDa; Ub, CS3936; HNE, Cell Biolabs STA-034; MG, Cell Biolabs STA-011; CML, Ab27684; AGE, Millipore 9890; Acetyl-lysine, CS9814.

## Results

In the brain, a subset of proteins that accumulate with age was first defined in flies as those that are insoluble in RIPA buffer and isolated by high-speed centrifugation [21]. Because of the demonstrable relevance of this subset of cellular proteins to lifespan and disease [1–3, 5, 6], we asked if an analogous set of detergent-insoluble aggregates could be identified in human brain tissue and if they changed in kind or amount with MCI and AD.

**Fig. 1:**
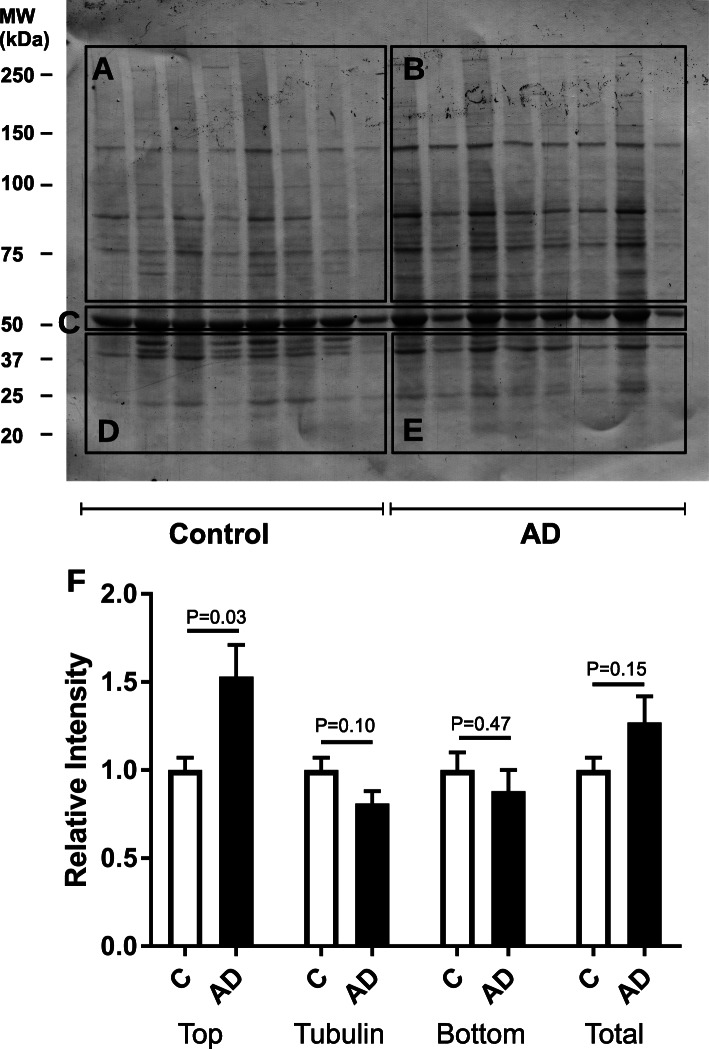
The AD brain has increased amounts of insoluble proteins. Amido black stained Western blot of control and AD cortex RIPA-insoluble fractions (aggregates). Each lane is a cortical sample from one individual, presented in the same order as the cases in Table S1 (1–16). The ratios reflecting the total protein in the various quadrants are presented along with tubulin. The 51 kDa protein (tubulin) was excluded from the analysis of each quadrant. *P* values are given. Data are presented as mean ± SEM (*n* = 8/group)

Approximately 100 mg blocks of cortical tissue from 8 AD patients and 8 age- (average 87 years) and sex-matched controls, along with 10 MCI patients and 10 age- (average 78 years) matched controls were extensively sonicated in 10 volumes of RIPA buffer and debris was removed by low-speed centrifugation. Insoluble molecules were pelleted at 81,000×*g* for 1 h, yielding soluble (supernatant) and insoluble (pelletome) fractions. Identical amounts of tissue from these pelleted fractions (based upon tissue weight of the samples) were run on SDS-polyacrylamide gels, transferred to a Western blotting membrane and stained with Amido black (Fig. 1). The AD pelletome blot was divided into 4 sections with the most abundant protein, the microfilament component tubulin (51 kDa molecular weight) forming the horizontal division (Fig. 1c). The amount of tubulin in the control and AD groups was quantified and excluded from the analysis of the 4 quadrants. When the lanes in each quadrant were scanned and quantified independently, there was no difference in the amount of protein between the lower molecular weight control and AD groups (sections D and E, respectively). However, there was a significant increase in the higher molecular weight proteins above tubulin in the AD patients (Fig. 1 a vs b). These differences were not observed in the soluble samples (Fig. S1). The same pattern was seen in the MCI soluble and pelletome fractions relative to their age-matched controls (Fig. S2 and S3).

### A subset of insoluble proteins is elevated in AD brain

To determine the identity of the RIPA-insoluble proteins enriched in the AD brain, the pellets and their supernatants were digested with trypsin and subjected to proteomic analysis. Two distinct approaches were used to determine the relative distribution of individual proteins in the pelletome fraction between AD and control tissue. In the first, the ranking from the proteomics data was based solely upon spectral counts, an approximation of abundance when the same proteins are compared [22]. The average spectral counts of individual proteins in the AD group were then divided by those from the control group, giving a ratio of the relative distribution of each protein.

For the second approach, the amount of each protein in the pellet fraction from each patient sample was first divided by the amount of the same protein in the supernatant fraction from the same patient before determining the AD to control ratio using spectral counts. The rationale for doing this was that the protein content may vary between individuals. This procedure thus normalizes the data for the initial individual protein content in each piece of tissue. The proteomics data were then verified by Western blot analysis.

Table 1 shows the top-ranked proteins using the first method, along with a few additional proteins of interest that were ranked slightly lower. The full list of proteins in both fractions is given in Supplementary Table S3. The top proteins included enzymes, structural proteins, and various members of the ubiquitin proteasome pathway (UPP). Calcium/calmodulin-dependent protein kinase 2 gamma (CAMK2) is a major protein involved in synaptic transmission, while glucose-6-phosphate isomerase (GPI) is a glycolytic enzyme. Coronin and dynamin are both actin-binding proteins. Enriched UPP proteins included polyubiquitin (UBB and UBC), ubiquitin ribosomal fusion protein UBA52, ubiquitin-like modifier activating enzyme UBA1, and two ubiquitin carboxyl-terminal hydrolases: USP14 and UCHL1/PARK5.

**Table 1:** Proteins more abundant in AD relative to control patients. Proteins in the same amount of 8 control and 8 AD cortical samples were determined by LC/MS/MS, generating spectral count data that reflects the relative amount of protein when two identical proteins are compared. The data are presented as the ratio of the spectral counts in AD divided by the control samples. A Wilcoxon rank-sum *t* test (on raw values) and a Welch’s t-test (on log-transformed quantile-normalized values) were performed to assess statistical significance between the two groups

Protein name	Ratio AD/control	Adjusted ***P*** value rank ***t*** test	Adjusted ***P*** value, log/quant ***t*** test	Description
CAMK2G	34.1	0.35284	0.36965	Calcium/calmodulin-dependent protein kinase type II subunit gamma
VARS	7.5	0.02652	0.02652	Valine-tRNA ligase
CORO1A	6.4	0.00292	0.01072	Coronin
UBC	6.1	0.18882	0.16372	Polyubiquitin-C
APOE	6	0.24853	0.25648	Apolipoprotein E
CADPS	5.9	0.02406	0.02181	Calcium-dependent secretion activator 1
UBB	5.9	0.18882	0.16481	Ubiquitin
UBA52	5.8	0.07478	0.05029	Ubiquitin-60S ribosomal protein L40
FN1	5.5	0.27685	0.26619	Fibronectin
CRMP1	5.4	0.15919	0.15661	Dihydropyrimidinase-related protein 1
GPI	5.3	0.04530	0.02897	Glucose-6-phosphate isomerase
USP14	5.2	0.05072	0.05530	Ubiquitin carboxyl-terminal hydrolase 14
DNM1L	5.1	0.04185	0.06481	Dynamin-1
DBNL	5.1	0.18936	0.22434	Drebrin
TOMM70A	5	0.12050	0.11536	Mitochondrial import receptor subunit TOM70
UBA1	4.9	0.06626	0.08523	Ubiquitin-like modifier-activating enzyme 1
SND1	4.6	0.02578	0.03000	Staphylococcal nuclease domain-containing protein 1
NNT	4.6	0.03093	0.02652	NAD(P) transhydrogenase, mitochondrial
XRCC6	4.3	0.04042	0.05530	KU-70
DLAT	4.2	0.02720	0.02652	Acetyltransferase component of pyruvate dehydrogenase complex
ACOT7	4.1	0.15434	0.16372	Cytosolic acyl coenzyme A thioester hydrolase
PPP2R4	4.1	0.02578	0.02652	Serine/threonine-protein phosphatase 2A activator
PLXNA1	4.0	0.08108	0.10703	Plexin-A1
PGRMC1	3.9	0.02447	0.01072	Membrane-associated progesterone receptor component 1
VPS35	3.8	0.04996	0.04909	Vacuolar protein sorting-associated protein 35
ATP2A2	3.8	0.04735	0.03000	Sarcoplasmic/endoplasmic reticulum calcium ATPase 2
KIF2C	3.6	0.02777	0.03000	Kinesin
MAPK3	3.6	0.02082	0.02652	Mitogen-activated protein kinase 3
HSPA12B	3.5	0.53236	0.52338	Heat shock 70 kDa protein 12B
RTN4	3.2	0.04345	0.07768	Reticulon-4
ARHGDIA	3.2	0.08045	0.09469	Rho GDP-dissociation inhibitor 1
FASN	3	0.08108	0.02652	Fatty acid synthase
XRCC5	2.9	0.05787	0.11224	KU-80
UCHL1	2	0.08705	0.03192	Ubiquitin carboxyl-terminal hydrolase isozyme L1

The analysis of the detergent-insoluble proteins using the second method yielded some of the same proteins, but their relative abundance was different and there were quite a few new proteins identified that were not found in the list shown in Table 1. Table 2 shows that the top protein is another actin binding protein called gelsolin, followed by the previously identified fatty acid synthase (FASN) and GPI. The previously identified DNA damage repair enzymes KU70 and KU80 are also included along with UBA1 and USP14. The full list is in Table S4.

**Table 2:** Proteins more abundant in AD relative to control patients, when both are first normalized to their respective soluble fraction. The ratio of AD to control is presented. A Welch’s *t* test was performed to assess statistical significance between the two groups

Protein name	Ratio AD/control	Adjusted ***P*** value	Description
GSN	3.2	9.71E−05	Isoform 3 of gelsolin
FASN	2.6	1.97E−05	Fatty acid synthase
XRCC6	2.4	1.71E−06	KU70
GPI	2.2	0.0004	Glucose-6-phosphate isomerase
CADPS	2.1	0.0005	Calcium-dependent secretion activator 1
UBA1	1.9	2.18E−05	Ubiquitin-like modifier activating enzyme 1
XRCC5	1.9	1.90E−06	KU-80
PGAM	1.9	3.97E−05	Phosphoglycerate mutase 1
HSP90AB1	1.8	8.60E−05	Heat shock protein HSP90-beta
ATIC	1.8	1.95E−05	Bifunctional purine biosynthesis protein PURH
HSP90AA1	1.8	0.0002	Heat shock protein HSP90-alpha
CKB	1.7	2.40E−05	Creatine kinase B
OGDHL	1.7	0.0005	2-oxoglutarate dehydrogenase-like, mitochondrial
HNRNPA2B1	1.7	0.0005	Heterogeneous nuclear ribonucleoproteins A2/B1
VPS35	1.7	0.0003	Vacuolar protein sorting-associated protein 35
RAP1GDS1	1.7	5.22E−06	Rap1 GTPase-GDP dissociation stimulator 1
CORO1A	1.6	0.0005	Coronin
DBNL	1.6	9.72E−05	Drebrin
USP14	1.6	0.0026	Ubiquitin carboxyl-terminal hydrolase 14

### Glycolysis is the predominate biological process associated with AD-related changes in insoluble proteins

To determine in more detail the association of the detergent-insoluble proteins with specific biological processes, we used a bioinformatics approach to analyze the data. For this analysis, we used the proteomics data based upon the ratio of pellet-to-soluble protein, as described in Table 2, because it may be a better method to normalize tissue variation within brains of different individuals than the ratio of spectral counts alone. The total proteomics data was used as opposed to only the limited proteins shown in the tables and figures. It was first asked whether or not there were significant differences between the soluble and pelletome groups, and secondly, were the proteins that were enriched in the AD pelletome relative to control associated with any specific physiologically relevant process.

**Fig. 2:**
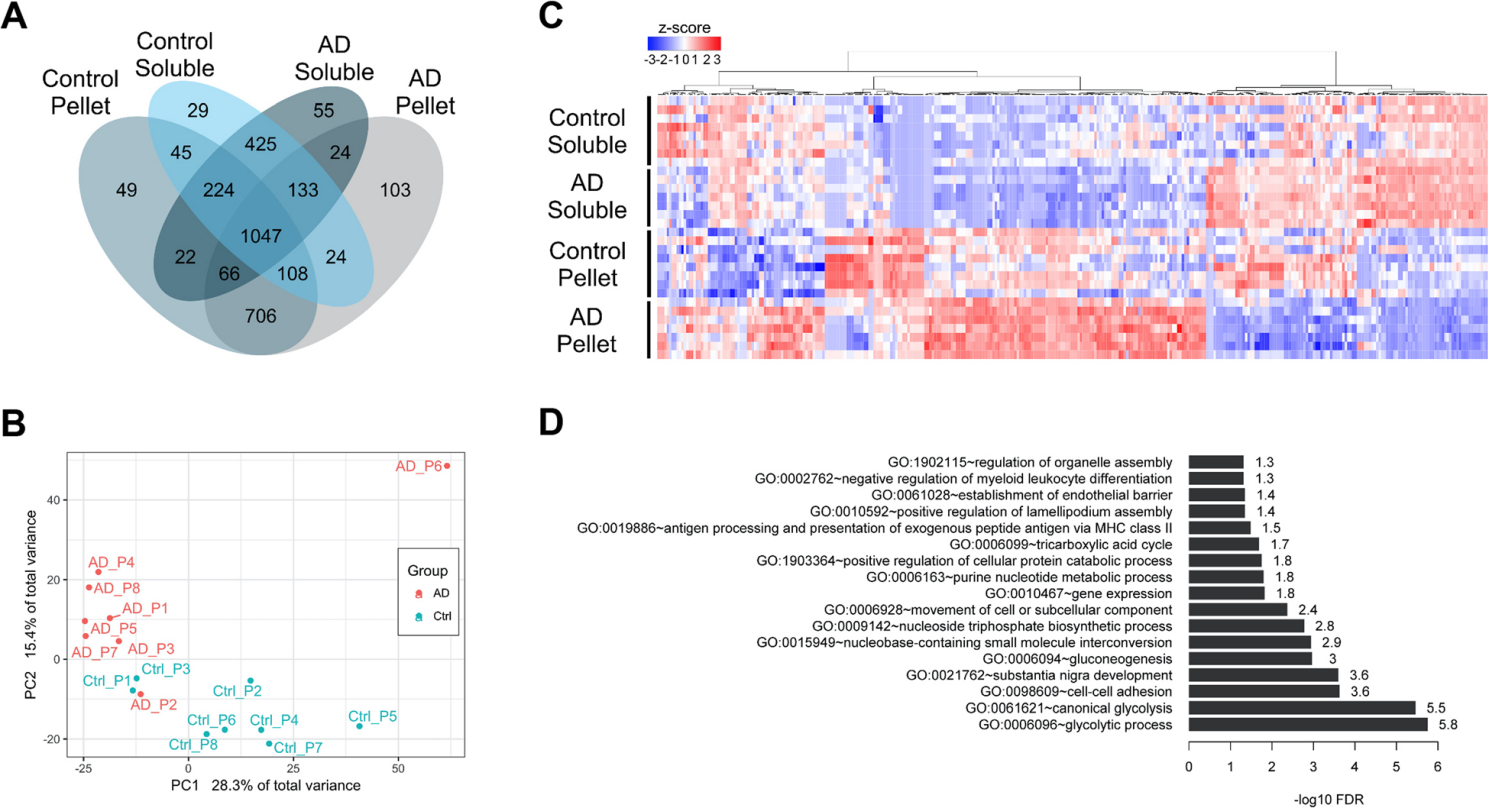
AD and control brains segregate into distinct clusters. Bioinformatic analysis of aggregated proteins in AD and control brains. **a** Venn diagram of expressed proteins (more than half of the biological replicates with non-zero counts) in soluble and pellet fractions of AD and control individuals. **b** Principle component analysis (PCA) plot of insoluble ratios for all proteins across individuals. Red = AD patients, blue = control patients. **c** Heatmap of 342 proteins with significantly different insoluble ratios between AD and control individuals. Values are *z*-scaled quantile normalized log-transformed spectral counts. Red = high, blue = low. **d** Bar plot of significantly enriched gene ontology (GO) biological process (BP) terms for the 342 proteins

A total of 3060 proteins were found in the soluble and pelletome fractions (Fig. 2a). Of these, the number of proteins in the soluble fractions was similar between AD (1996) and control (2035). Furthermore, the number of proteins in the pelletome fractions was also similar between AD (2211) and control (2267). For each sample, an insoluble ratio per protein was calculated based on the log fold difference between its pelletome and soluble fractions. Principle component analysis (PCA) showed that the protein ratios segregated into distinct clusters, demonstrating distinct differences in the pelletome between the AD and control brains (Fig. 2b). The AD patient P6 (Patient 14, Table S1) was determined to be an outlier based on the PCA plot and was excluded from further analysis. The pellet-to-soluble ratios were then compared between patient groups using a two-tailed *t* test. The heatmap in Fig. 2c of proteins with significantly different insoluble ratios between AD and control individuals shows that many of the pelletome proteins increased with AD. While there were differences between AD and control proteins in the soluble fractions, they were much fewer. In most individuals, there was an inverse relationship between the levels of the proteins in the soluble and insoluble fractions. When the proteins differentially accumulated in the AD pelletome were examined in a pathway analysis paradigm, the top two GO biological processes were associated with glycolysis (Fig. 2d). There is extensive literature, including work from our own lab, showing that glycolytic function is decreased with aging, and more rapidly in AD patients [23–25].

### Western blotting of AD brain supports proteomics data

To verify the proteomics data, the same fractions were analyzed for protein levels by Western blot using antisera against a number of the top proteins most abundantly found in the AD pelletome. This subset of proteins was also examined by Western blot in the MCI samples. The quantitation for each set is shown in Figs. 4, 5, and 6, and the blot profiles for all samples are shown in the Supplementary Material. Because there was no obvious internal loading control in the pelletome fractions, all samples were first run on Bio-Rad Stain-Free gels in which a trihalo compound inside the gel binds to proteins, allowing quantification of the total amount of protein in each lane when the blots are scanned on a Bio-Rad ChemiDoc MP Imaging System. The total protein for each lane of the blot membrane was then used to normalize the Western blot data as is frequently done with actin or housekeeping enzymes. The proteins in the figures are grouped roughly according to function. A few additional proteins of biological interest were also included in the Western blot studies. Importantly, the Western blot data adds new insight into these proteins by allowing comparison between the AD and MCI brains.

**Fig. 3:**
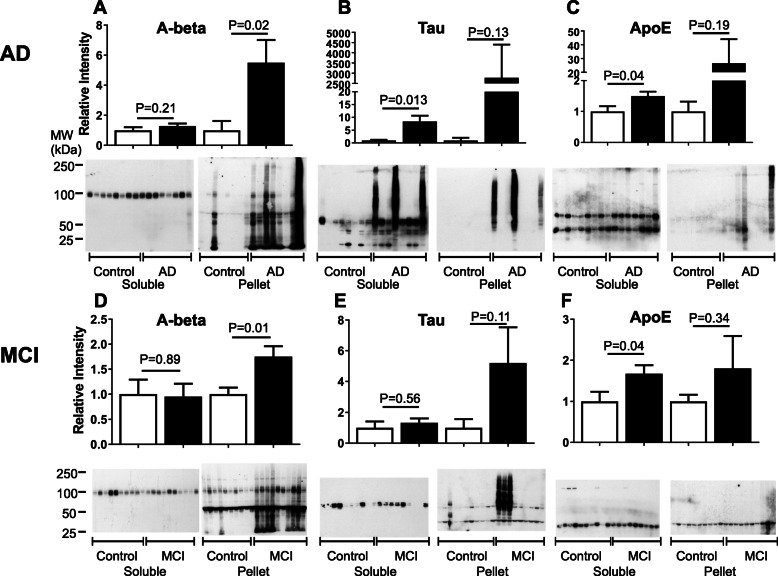
Increased levels of disease-associated proteins in AD and MCI brain pelletomes. Western blots and quantifications of disease-associated proteins. **a** Aβ (6E10 antibody) AD. **b** (pan) Tau AD. **c** (pan) ApoE AD. **d** Aβ (6E10 antibody) MCI. **e** (pan) Tau MCI. **f** (pan) ApoE MCI. The 100 kDa protein in **a** and **d** is APP. The 50 kDa protein in **d** is non-specific antibody recognition. *P* values are given. Data are presented as mean ± SEM (*n* = 8–10/group)

Three proteins directly related to classical AD pathology were also examined: Aβ, tau, and ApoE (Fig. 3). With the exception of one individual, none of the controls had much Aβ in their soluble or pelletome fractions. In contrast, aggregated Aβ was highly enriched in both the AD and MCI pelletome relative to controls. The 100 kDa protein is the amyloid precursor protein (APP) that is recognized by the 6E10 antibody. Tau was increased and highly aggregated in the soluble and pelletome fractions of three AD patients and in the pelletome fraction of 3 MCI patients (Fig. 3b, e). Phosphorylated tau at positions 396 and 404 was also elevated in these same individuals (not shown). There was little ApoE detected in the pelletome samples, but it was aggregated in two AD patients (Fig. 3c, f). However, ApoE was significantly elevated in the soluble AD and MCI fractions relative to the control patients. The molecular weight of monomeric ApoE is 34 kDa, but it tends to aggregate, explaining the 70 kDa band in the soluble fraction [26]. Therefore, using our methods, insoluble Aβ and soluble ApoE were correlated with the AD disease state, and both proteins showed increased levels in MCI as well.

**Fig. 4:**
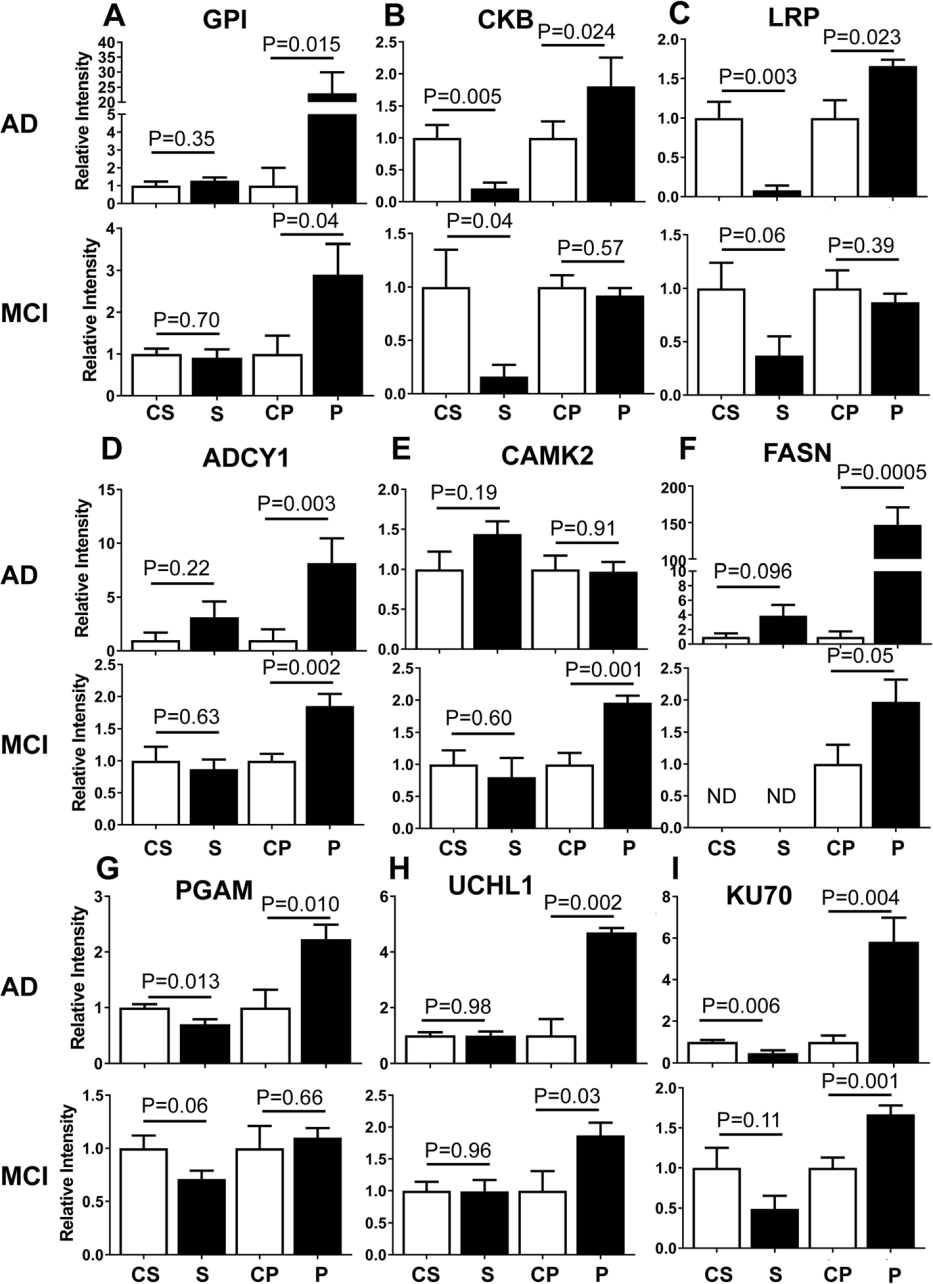
Increased levels of enzymes in AD and MCI brain pelletomes. Western blot quantifications of enzymes identified by proteomics. **a** Glucose-6-phosphate isomerase (GPI). **b** Creatine kinase B (CKB). **c** Low density lipoprotein receptor-related protein (LRP). **d** Adenylate cyclase isozyme 1 (ADCY1). **e** Calcium/Calmodulin protein kinase 2 (CAMK2). **f** Fatty acid synthase (FASN). **g** Phosphoglycerate mutase (PGAM). **h** Ubiquitin carboxyl-terminal hydrolase isozyme L1 (UCHL1). **i** KU70. C, control; S, soluble; P. pelletome. *P* values are given. Data are presented as mean ± SEM (*n* = 8–10/group)

Figure 4 shows that a number of the enzymes identified by proteomics to be elevated in the AD pelletome relative to age- and sex-matched controls could be confirmed by Western blot analysis. We also assessed additional proteins of interest, including low density lipoprotein receptor-related protein (LRP) and adenylate cyclase isoenzyme 1 (ADCY1). GPI, creatine kinase B (CKB), LRP, ADCY1, FASN, phosphoglycerate mutase 1 (PGAM), UCHL1, and KU70 were all increased in the AD pelletome samples. The Western blot data using a pan-CAMK antibody indicated that while CAMK2 was not elevated in the AD pelletome in contrast to the proteomics data, it was increased in the MCI pelletome. In the case of CKB, LRP, PGAM, and KU70, there was a corresponding decrease of the protein in the soluble fractions (Fig. 4). Curiously, there were also several low molecular weight forms of KU70 found in both the AD and MCI brains (Figs. S4 and S5).

Significant differences were observed between AD and MCI in about half of these enzymes (Figs. 4, S4, and S5). Proteins that were significantly enriched in the pelletome of both AD and MCI included GPI, ADCY1, FASN, UCHL1, and KU70. Proteins with reduced levels in the soluble fraction of both MCI and AD included CKB and LRP, but only in the AD samples did these proteins increase in the pelletome. All together, these data show that many of the shifts in protein solubility that occurred within this subset of proteins in AD were also found in MCI patients.

**Fig. 5:**
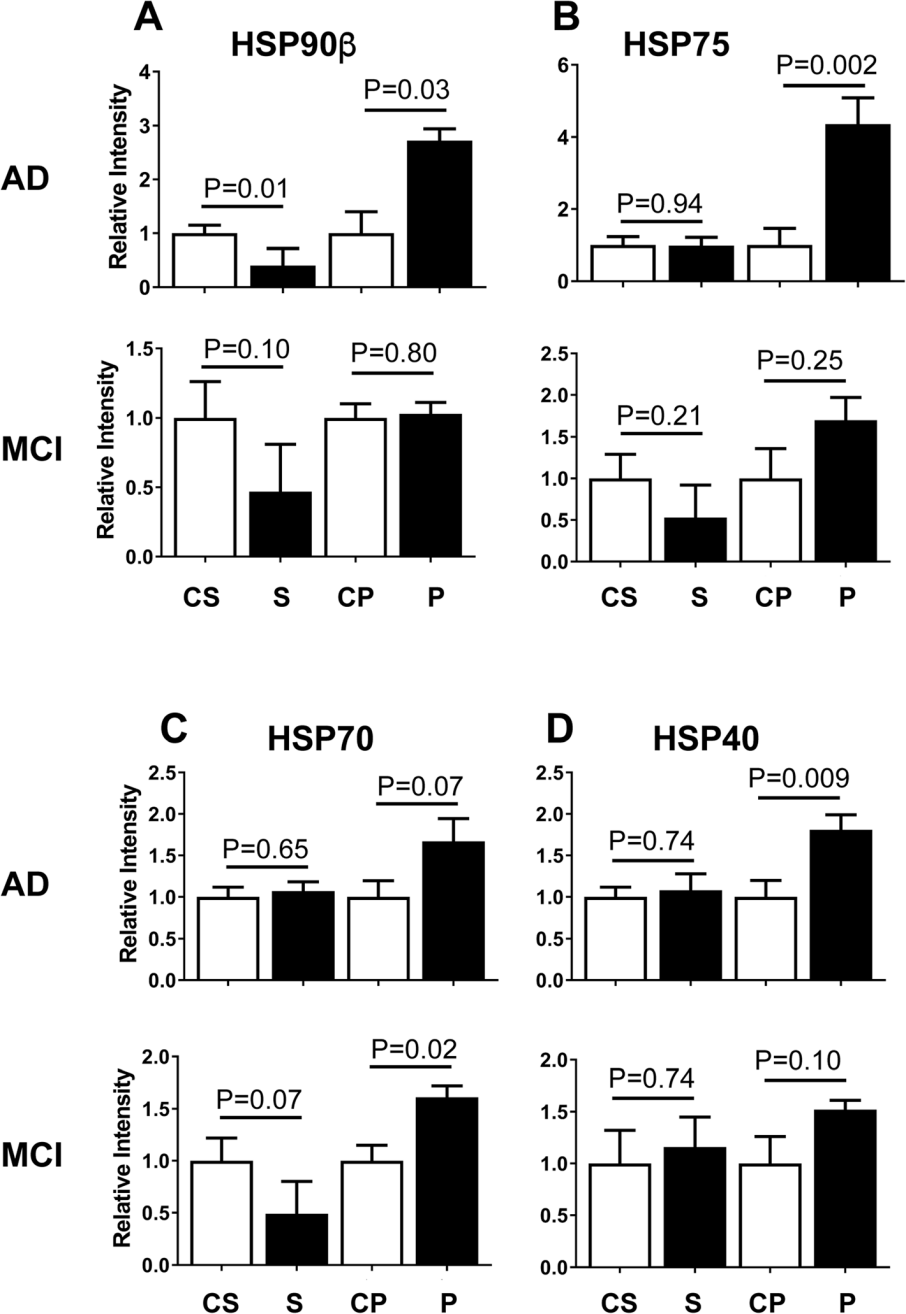
Increased levels of heat-shock proteins in AD and MCI brain pelletomes. Western blot quantifications of heat-shock proteins. **a** HSP90β. **b** HSP75. **c** HSP70. **d** HSP40. C, control; S. soluble; P, pelletome. *P* values are given. Data are presented as mean ± SEM (*n* = 8–10/group)

Proper protein folding is required for proteins to achieve functional activity, and misfolding frequently leads to aggregation. Folding is assisted by a large family of chaperone proteins, of which heat shock proteins are a subset. Four heat shock proteins were examined by Western blot in AD and MCI brains: HSP90β, HSP75, HSP70, and HSP40. These as well as other HSPs are thought to be involved in the pathogenesis of AD and many are viewed as potential therapeutic targets for the disease [27]. Figures 5, S6, and S7 show that all of these proteins were elevated over controls in the AD pelletome, but only HSP70 was enriched in the MCI pelletome. HSP70 is of particular interest because of its ability to inhibit and solubilize aggregates of Aβ and tau [28]. The observation that HSP70 becomes detergent-insoluble in the MCI brain before full-blown AD pathology suggests that it may be a valid therapeutic target. Furthermore, enrichment of HSP90β in the AD pelletome is consistent with the proteomic analysis (Table 2).

**Fig. 6:**
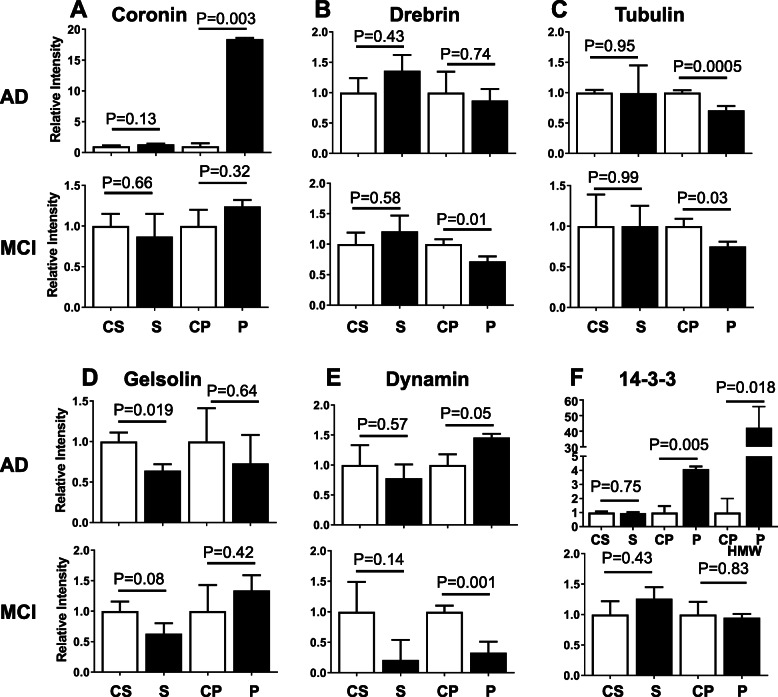
Altered levels of structural proteins in AD and MCI brain pelletomes. Western blot quantifications of structural proteins identified by proteomics. **a** Coronin. **b** Drebrin. **c** Tubulin. **d** Gelsolin. **e** Dynamin. **f** 14-3-3. For the AD pelletome fraction, 14-3-3 was observed at both its predicted molecular weight (28 kDa) and at 220 kDa (HMW). C, control; S, soluble; P. pelletome. *P* values are given. Data are presented as mean ± SEM (*n* = 8–10/group)

There were also a number of structural proteins that showed altered levels in AD and MCI (Figs. 6, S8, and S9). The most significant of these was coronin, which was much more highly concentrated in the AD pelletome relative to MCI. Drebrin levels were decreased in the MCI pelletome, but not AD. Tubulin showed decreased levels in both the AD and MCI pelletome, consistent with previous studies of postmortem brain tissue from AD patients [29]. Gelsolin levels were similar in AD and MCI. The structural protein dynamin was increased in the AD pelletome, but decreased in the MCI pelletome. 14-3-3, a low molecular weight regulatory protein that binds a large number of other proteins [30], was highly abundant in the AD pelletome, but not enriched in MCI. This protein was also detected at 220 kDa by Western blot (Figs. 6f HMW, S8F HMW).

### Secondary protein modifications have altered levels in AD brain

Proteins can be modified by the addition of various small molecules that affect their activity, structure, and aggregation [31]. Because of its role in protein degradation via the ubiquitin pathway, the modifications associated with lysine are of particular interest. Additionally, several lysine modifications also lead to protein crosslinking [32]. Lysine residues can be modified by a number of processes besides ubiquitination, including acetylation [33] and glycation. Glycation leads to a variety of protein modifications generically classified as advanced glycation end products (AGEs) that can be identified by antisera against AGEs (all modifications), or the more specific carboxymethyl-lysine (CML) and methylglyoxal (MG) adducts. MG is a highly reactive dicarbonyl that is a byproduct of glucose metabolism and lipid peroxidation that, like ubiquitin, can cross-link proteins [34]. Another lysine-associated secondary modification that we examined was 4-hydroxynonenal (HNE), a product of lipid peroxidation [35]. HNE also modifies proline in addition to lysine. All of these modifications increase overall protein hydrophobicity and enhance the risk for aggregation [36]. In addition, because these modifications of lysine are covalent, they could compete with each other to determine the fate of the protein.

**Fig. 7:**
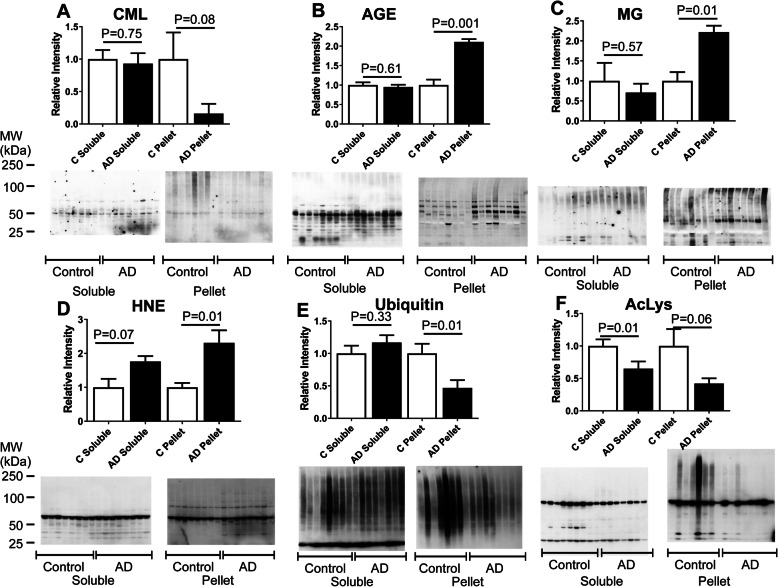
Altered levels of secondary protein modifications in AD brain pelletome. Western blots and quantifications of protein secondary modifications. **a** Carboxymethyl-lysine (CML). **b** Advanced glycation end products (AGE). **c** Methylglyoxal (MG). **d** 4-hydroxynonenal (HNE). **e** Ubiquitin. **f** Acetyl-lysine (AcLys). *P* values are given. Data are presented as mean ± SEM (*n* = 8/group)

To gain insight into the modification of proteins in the AD and MCI cortex, 6 potential modifications at this site were examined (Figs. 7 and S10). CML was significantly decreased in the MCI pelletome and non-significantly decreased in the AD pelletome (Figs. 7a and S10A). In contrast, AGE formation, defined by antiserum prepared against glucose glycated albumin, was greatly increased in the pelletome of both AD and MCI (Figs. 7b and S10B). Two markers of oxidative and electrophilic stress, MG and HNE, respectively, were both elevated in the AD pelletome, but not in the MCI pelletome (Figs. 7c and d, and S10C and D). Finally, two major modifications directly involved in cell signaling, ubiquitin and acetyl-lysine, were both reduced in the AD pelletome, but only acetyl-lysine was reduced in the MCI pelletome (Figs. 7e and f and S10E and F). While ubiquitin mediates protein degradation, acetyl-lysine modifications directly reflect the level of acetyl-CoA in cells [37]. It has recently been shown that acetyl-CoA is reduced in mouse models of aging and AD, and is elevated to basal levels by two AD drug candidates that have therapeutic and anti-aging effects in these mice [38]. Therefore, it is possible that a dynamic interaction exists between the loss of protein acetylation and ubiquitination and the increase in other more toxic modifications with age that lead to protein insolubility.

### Reduced protein solubility in MCI is further exacerbated in AD

**Fig. 8:**
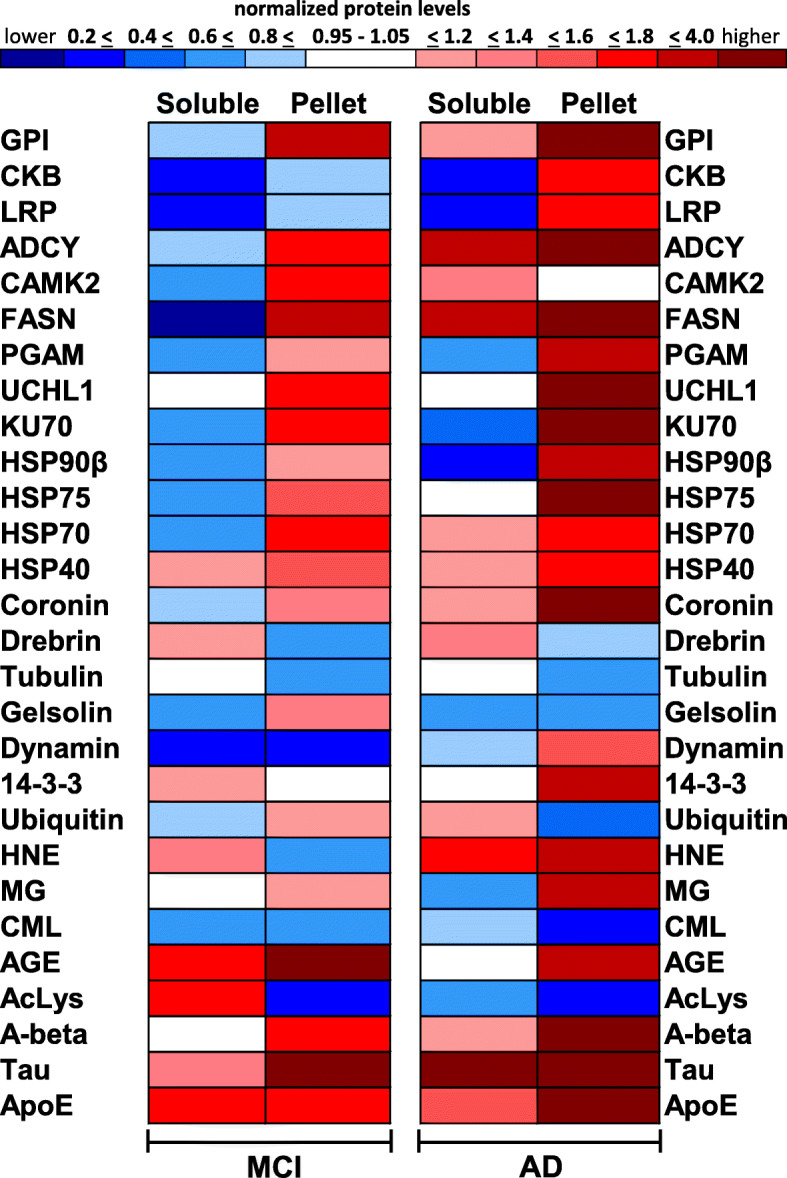
Protein level alterations in brain increase with progression from MCI to AD. Heatmap of protein levels in soluble and pellet fractions of MCI and AD patients. Each protein analyzed was first normalized to total protein and then to its level in the control group. Blue represents decreased protein level, red represents increased protein level, and white represents no change in protein level when compared to the control group. Mean data are presented (*n* = 8–10/group)

To better visualize the alterations in protein levels that occur in the soluble and pelletome fractions of MCI and AD patients, the Western blot data were reformatted in the form of a protein level heatmap (Fig. 8). Each protein analyzed was first normalized to total protein and then to its level in the control group (as 1 Relative Level) to allow for a direct comparison of changes in protein levels between MCI and AD. Deviations from 1 in the positive direction (increased abundance) are denoted by increasing shades of red, while deviations from 1 in the negative direction (decreased abundance) are denoted by increasing shades of blue. A 5% or less deviation from 1 in either direction is denoted by white and could be considered as no change relative to the control group. The technique of normalizing each protein to its level in the control group is performed so a visual comparison can easily be made between MCI and AD with regard to progression-dependent protein level alterations. We used a color-coding system that changes intensity for each 20% change in normalized protein level, up to a 2-fold change.

Extreme differences are represented by darker colors and moderate differences are represented by lighter colors. For example, in the soluble fraction of MCI patients, CKB levels were decreased by 80% relative to the control group and the band is colored dark blue. In the pelletome fraction of MCI patients, CKB levels were decreased by 10% relative to the control group and the band is colored light blue. However, in the pelletome fraction of AD patients, CKB levels were increased by 70% relative to the control group and the band is colored red. This technique loses resolution for highly enriched proteins such as tau in the AD cohort. Here we see that tau was increased 10-fold compared to controls in the soluble fraction and 3000-fold compared to controls in the pelletome fraction. However, because we set our upper limits of color differentiation at 4-fold enrichment, both of these boxes are colored dark red.

MCI- and AD-induced protein level alterations were generally more extreme in the AD cohort, as denoted by the darker colors (Fig. 8). In the soluble fraction of MCI patients, the levels of 8 proteins increased, 16 decreased, and 4 remained unchanged. In the MCI pelletome fraction, the levels of 19 proteins increased, 8 decreased, and 1 remained unchanged. A similar pattern was seen in the AD cohort, but with a greater shift towards increased protein levels. In the AD soluble fraction, the levels of 13 proteins increased, 10 decreased, and 5 remain unchanged. In the AD pelletome fraction, the levels of 21 proteins increased, 6 decreased, and 1 remained unchanged. In summary, the pattern of protein level changes in the pelletome of MCI patients was generally mirrored in AD, with more extreme changes occurring in the AD cohort.

To better understand how the disease-associated proteins (Aβ, tau, ApoE) may correlate with the accumulation of other proteins in the pelletome fraction, we performed pairwise correlation analyses (Tables S5 and S6). The only significant correlation found in MCI was between Aβ and HSP70 (*r* = − 0.608, *p* = 0.031), and this became a positive correlation in AD (*r* = 0.703, *p* = 0.026). Within the AD cohort, HSP70 was also correlated with tau (*r* = 0.806, *p* = 0.0079) and nearly reached significance with ApoE (*r* = 0.612, *p* = 0.053). Aβ and ApoE were highly correlated (*r* = 0.948, *p* = 0.00017). Coronin was correlated with Aβ (*r* = 0.641, *p* = 0.043), tau (*r* = 0.635, *p* = 0.045), and ApoE (*r* = 0.645, *p* = 0.042). ADCY1 was correlated with both Aβ (*r* = 0.680, *p* = 0.032) and ApoE (*r* = 0.680 *p* = 0.032). Additionally, tau was correlated with GPI (*r* = 0.811, *p* = 0.0072) and HSP75 (*r* = 0.664, *p* = 0.036).

### Aggregation of low molecular weight proteins

Some proteins, such as Aβ, can form high molecular weight aggregates that remain intact in the presence of SDS without covalent cross-linking, and migrate as high molecular weight aggregates in SDS-PAGE (Fig. 3a). This is frequently due to the formation of structures held together by β-sheets [39]. Therefore, we asked if similar aggregates of additional proteins in the AD brain could be identified. This was done by running detergent-insoluble pellets of control and AD cortex on SDS-PAGE and determining which proteins migrated on the gels at molecular weights significantly higher than their predicted values and were also present at higher levels in the AD brain.

Samples of AD and age- and sex-matched controls were run in parallel lanes on SDS-PAGE, stained with Coomassie blue, and each lane was cut into 10 fractions at the identical molecular weight levels. Each excised piece of gel was extracted with urea and the proteins were digested with trypsin. Figure S11 shows the staining pattern of the gel along with the molecular weight standard. The proteins in each pair of the 10 fractions were analyzed by LC/MS/MS and ranked by the ratio of the amount of protein in AD over control tissue. Supplementary Table S7 shows the proteins identified in fraction 1 which migrate above 200 kDa but have a predicted molecular mass of tubulin (51 kDa) or less. The complete list of proteins in polyacrylamide gel fractions 1 through 10, ranked according to spectral counts, is in Supplementary Table S8.

Eleven proteins in this list were increased over 50-fold in the AD pelletome when compared to controls, and included UCHL1, a protein identified by the proteomics analysis (Table 1) and validated by Western blot in both AD and MCI brains (Fig. 4h). Additionally, dual specificity mitogen-activated protein kinase kinase 1 (MAP2K1) was also increased over 50-fold in the AD pelletome. This is of interest because MAP2K1 is part of the mitogen-activated protein kinase (MAPK) signaling cascade, which is involved in a variety of crucial cellular and physiological processes including cell proliferation, differentiation, adhesion, migration, and survival [40]. ADCY1, a neurospecific protein enriched at the postsynaptic density, was also increased over 50-fold and validated by Western blot in both AD and MCI brains (Fig. 4d). Another abundant protein migrating well above its predicted molecular weight was 14-3-3 (predicted mw = 27–29 kDa). This protein was also detected at 220 kDa by Western blot (Figs. 6f, S8). 14-3-3 has 7 isoforms with a multitude of functions, and it interacts with a variety of proteins. 14-3-3 proteins are associated with tubulin and tau, as well as neurofibrillary tangles [41]. Importantly, many of the proteins migrating well above their predicted molecular weights were also enriched in the AD pelletome fraction. However, the majority of the proteins migrated in the SDS-polyacrylamide gels as predicted by their molecular weights (Table S8).

## Discussion

Proteostasis maintains the tightly regulated balance between gene expression, quality control, and protein degradation. As organisms age, this balance becomes disrupted and there is an accumulation of aggregated proteins, many of which become highly insoluble [1, 2, 36]. While this occurs in all organisms, the accumulation and aggregation of specific proteins are amplified in neurodegenerative diseases such as AD (Aβ) and Parkinson’s (α-synuclein), and considered hallmarks of these diseases. However, additional proteins also aggregate with age and may contribute to disease development and/or progression. Here we show that of a subset of proteins defined as those that are detergent-insoluble and pelleted by high-speed centrifugation were increased in the cortical brain tissue of AD patients relative to age- and sex-matched controls and that many of these same changes were also seen in brains from MCI patients. Because it is not possible to rigorously define the aggregation state of a protein by detergent solubility alone, we operationally call these proteins the pelletome.

While the solubility characteristics of the canonical disease-associated proteins have been well studied, additional detergent-insoluble proteins that aggregate in the brain are not as well characterized. There has been a thorough study of these proteins in transgenic AD mice by comparing phosphate buffered saline (PBS)-soluble and SDS-insoluble fractions. An analysis of the data revealed that of the top 34 SDS-insoluble proteins from the transgenic tau model, only 7 are shared with our human data (UBA1, FASN, GPI, RAP1GDS1, PGAM, VPS35, and HSP70) [12]. However, a bioinformatics study of these data identified nodes that were similar to what we observed, including proteins involved in energy metabolism, cytoskeletal proteins, and hydrolases [12].

There have also been several studies examining both total and membrane proteins from control, asymptomatic AD (cognitively normal people with plaques and tangles) and AD patients by mass spectrometry followed by network analysis. In general, the subset of total proteins found to change in AD did not overlap with the top proteins we found in the pelletome of AD patients, but there were overlaps on the modules identified by network analysis [9, 10]. These include those associated with mitochondrial energy metabolism in addition to glial cells and inflammation. Glycolysis was also a major network analysis module when only membrane proteins were assayed [42].

More directly relevant to our data are several studies with human tissue that examined detergent-insoluble proteins in human AD brain, but using different extraction protocols. A study of human AD and control cortical samples that were sequentially extracted with Triton X-100 and sarkosyl yielded a set of proteins that were, with the exceptions of 14-3-3, dynamin, ApoE, tau, and Aβ, largely distinct from those in mice and from our study [13]. A sequential study of AD development from control and MCI to symptomatic AD correlated nearly 3000 sarkosyl-insoluble proteins with the expression of Aβ and tau, and found that the best correlation was with small ribonucleoproteins [14], a set of proteins we failed to identify. But pathway and cellular component analysis identified proteins involved in energy metabolism as a major component of the insoluble fractions. Finally, another study analyzed Triton X-100 and sarkosyl-insoluble proteins from controls and demented subjects that were highly aggregated and migrated above their native molecular weights in SDS-PAGE. The most enriched proteins in that fraction were tau, TDP-43, alpha-synuclein, and ApoE [43]. In our data set, tau was enriched in the AD fraction migrating above 200 kDa, but the others were not detected in meaningful quantities (Table S8).

Using a different protocol from those above, we were able to identify another set of detergent-insoluble proteins that were differentially concentrated in the cortex of AD and MCI patients compared to their respective controls. Group-specific secondary modifications were also found, showing that proteins were modified differently in the AD and MCI brain. We also identified a significantly overrepresented gene ontology biological process associated with the alteration of protein aggregation between AD and control patients.

It is important to note our workflow reasoning in this project. Because spectral counts are an approximation of abundance when the same proteins are compared [22], we used the spectral count rankings only as a guide to determine which proteins to validate with Western blot, instead of an absolute determination of significance. We were first interested in the raw rankings (Table 1). Due to the non-normal distribution inherently observed when assessing spectral counts across variable biological samples, we decided it would also be useful to rank the data following multiple normalizations (Table 2). We then focused our validation efforts on proteins that intersected both lists or were ranked near the top of either list.

With regard to the enzymes shown in Fig. 4, we observed increased levels in the pelletome fraction of AD patients for GPI, CKB, LRP, ADCY1, FASN, PGAM, UCHL1, and KU70. Increased levels of CAMK2 were not observed in contrast to the mass spectrometry data (Table 1); however, a significant increase was seen in the MCI pelletome fraction. This discrepancy could be due to our use of a pan-CAMK2 antibody (the gamma subunit was enriched in Table 1). GPI, PGAM, and CKB have important functions in brain energetics, with the former two acting as glycolytic enzymes and CKB acting as a kinase in the phosphocreatine circuit. LRP is a transmembrane endocytic receptor that is highly expressed in neuronal cell bodies and neurites [44]. LRP binds ApoE as well as Aβ and is genetically linked to AD [44]. While LRP is not a bona fide enzyme, we included it in this category because of its implications in signal transduction [45]. ADCY1 is a neurospecific calcium/calmodulin-regulated adenylate cyclase implicated in learning, memory, and behavior [46]. FASN is a multifunctional protein whose main function is to synthesize the fatty acid palmitate from acetyl-CoA and malonyl-CoA [47]. As these proteins become insoluble, they likely exhibit decreased/lost functionality for a diversity of critical cellular processes.

KU70 (Figs. 4, S4, and S5 and Tables 1 and 2) and KU80 (Tables 1 and 2) should also be noted. KU70 and KU80 make up a heterodimer that is a major enzyme involved in DNA damage repair and the maintenance of telomere length. Mice homozygous for KU70 mutations are very sensitive to ionizing radiation [48], and mice lacking the KU70 or KU80 gene are characterized by premature aging [49]. Therefore, it has been argued that double-strand DNA breaks contribute to aging, and it is known that DNA damage repair is reduced in the AD brain [50]. Our data show that KU70 became insoluble in both the AD and MCI brain and was also fragmented in the AD and MCI pelletome fractions. It was additionally reduced in the soluble fraction of the AD brain.

There was an increased abundance of UCHL1 in both the AD and MCI pelletome. The UPP is a major mechanism for the removal of abnormal proteins, which is critical for the survival of neurons. Before a ubiquitin-tagged protein enters the proteasome, ubiquitin is removed by a deubiquitinating enzyme such as UCHL1. Additionally, UCHL1 also functions as an E3 ubiquitin ligase. Deficits in ligation/hydrolysis of ubiquitin to/from proteins negatively impact the functionality the UPP and will likely have amplified downstream consequences with regard to proteostasis [51]. Previous studies have shown UCHL1 (PARK5) to co-aggregate with α-synuclein in Lewy bodies of Parkinson’s disease patients and aggregation/unfolding could be promoted by secondary modifications such as HNE [52]. We observed increased levels of HNE modification in the AD pelletome fraction (Fig. 7). We also observed decreased levels of ubiquitination in the AD pelletome fraction, suggesting deficits in UPP functionality (Fig. 7).

With regard to the structural proteins shown in Fig. 6, we observed increased levels in the pelletome fraction of AD patients for coronin, dynamin, and 14-3-3. However, gelsolin and drebrin did not show increased levels. For gelsolin, we again used a pan-antibody; however, only isoform 3 was enriched in the results shown in Table 2.

Coronin is an actin-binding protein that was only enriched in the AD pelletome. Coronin is abundantly expressed in microglia and is frequently used as a histochemical marker for these cells [53]. Increased levels of coronin in the AD pelletome could be due to a higher number of microglia associated with amyloid plaques in AD relative to MCI patients.

Dynamin is a GTPase that mediates outer mitochondrial membrane fission and inhibitors of the protein block cell death [54]. We observed decreased dynamin levels in the MCI pelletome, but increased levels in the AD pelletome. This may suggest a potential compensatory mechanism occurring in MCI that is overrun in the progression to AD.

14-3-3 proteins consist of a family of proteins that are highly expressed in the brain and impact most aspects of its function, including signaling, development, and neuroprotection [55]. These proteins bind to over 200 other proteins, some very tightly, and are enriched in the ultrastructure of synapses. Here we show using a pan-antibody that 14-3-3 proteins were enriched 4-fold in the AD pelletome relative to controls. In addition, 14-3-3 proteins were also found at a very high molecular weight on SDS-PAGE, migrating at over 200 kDa with a 40-fold enrichment relative to controls (Figs. 6f HMW, S8F HMW). Due to its large number of binding partners, reduced 14-3-3 solubility may have amplified downstream consequences with respect to brain functionality.

Aβ plaques are a hallmark of AD and feature the highly aggregated and profoundly insoluble Aβ peptide. Polymerized Aβ was highly enriched in the pelletome fraction of both AD and MCI cortex relative to controls, along with other proteins found in plaques micro-dissected from brains of AD patients. These proteins include HSP90β, coronin, tau, 14-3-3, and dynamin [56].

Neurofibrillary tangles (NFTs) are a feature of the AD brain as well as multiple forms of brain injury. Tau is a major component of NFTs, and the protein content of NFTs isolated by laser capture micro-dissection of AD patients has been determined [57]. Of the 72 proteins identified in NFTs, 28% were associated with metabolic functions. Using a bioinformatics approach based upon total proteomics data, we similarly showed that proteins involved in glycolysis were highly associated with the alteration of protein aggregation between AD and control patients. A number of the NFT-associated proteins matched what we found in the AD pelletome, including the expected structural proteins dynamin and tau.

It has been argued that tau pathology is a valid target for AD and a number of ongoing clinical trials are directed against it. Our data show that tau pathology in the context of its polymerization and insolubility was not associated with the majority of the AD patients in our cohort; however, those exhibiting pathology showed dramatically increased levels. Only 3 of the 8 AD patients had significant levels of polymerized tau, while all had high levels of Aβ in the pelletome fraction. Nevertheless, the AD cohort exhibited a 3000-fold enrichment of polymerized tau relative to cognitively normal controls. This was the most altered protein level observed in our study, although non-significant due to patient variability. These data suggest that caution and/or more refined measures are needed when using tau as a diagnostic tool or drug target for AD, as it may be useful in only about half of the AD cohort.

There has been an extensive amount of proteomics-based work on secondary modifications in the AD brain (for recent reviews, see [58, 59]). The majority of this work has focused on specific proteins such as tau and those in the amyloid pathway [60], but it is becoming very clear that additional proteins in the brain can be extensively modified at lysine residues. Of particular significance is work from the Butterfield laboratory showing that many proteins related to glycolysis and energy metabolism are oxidatively modified [61]. It is generally believed that oxidized and glycated proteins are more prone to aggregation [62, 63], and this was reflected in our data.

We assessed six modifications associated with lysine residues in proteins. Ubiquitination is a process that binds the ubiquitin peptide to lysine via an isopeptide bond, frequently leading to protein degradation. HNE is a byproduct of lipid peroxidation that forms a Michael addition product with lysine [64]. CML is formed from a Maillard reaction with lysine by the addition of the metabolic products glyoxal and glyceraldehyde [65]. Methylglyoxal is formed as a byproduct of normal metabolism and has many possible protein adducts [66]. All four of these modifications have the potential to covalently cross-link and aggregate proteins [63]. The generic term for the latter three is advanced glycation end products (AGEs). This group of modifications can also be created by the addition of sugars like glucose and fructose, and are recognized by an anti-AGE antiserum. Finally, lysine is often modified by acetylation and this can alter many physiological processes [33].

Our data show that in the AD pelletome there was a decrease in ubiquitination, acetylation, and CML-modification, while there was an increase in MG- and HNE-modification. It has been argued that glycation of proteins is unfavorable because it can lead to aggregation and loss of function. In contrast, ubiquitination aids in protein degradation, and protein acetylation likely has an overall positive influence because its reduction is associated with neurodegenerative disease [38]. Therefore, it can be argued that the glycation of lysine blocks the addition of ubiquitin or acetate, and is likely detrimental to overall cell physiology. Indeed, since protein glycation and HNE modifications increase with aging in all animals, some have argued that these modifications may be the cause of age-related morbidities [63, 64, 67]. Our data support the possibility that glycation or HNE addition may interfere with the normal removal and/or degradation of proteins, leading to their aggregation and functional demise.

Finally, the most relevant question: how does the accumulation of detergent-insoluble proteins with aging and AD affect the brain and disease pathology? While most of the work in the AD field has focused on Aβ and tau, all aggregated proteins likely have a potential for toxicity. Individual proteins can form aggregates with multiple, very distinct conformations that are dependent upon the biological and physical events that lead to aggregation, while soluble proteins usually have only one conformation that impacts both solubility and biological activity [68]. When five different conformational states of the N-terminal domain of hydrogenase maturation factor were tested for neurotoxicity in the identical assays used to define Aβ toxicity, some of the aggregate structures were toxic, while others were not [68]. In addition, proteins are able to propagate and transfer radicals much like what occurs in lipid peroxidation, and this trait may be enhanced in misfolded proteins [69]. Since the AD brain is a pro-oxidant environment and oxidized proteins are more prone to aggregate, AD likely facilitates protein aggregation.

Protein structure and function are tightly linked properties. Manipulation of a protein’s structure via formation of complexes, post-translational modifications, or alternative splicing may confer a diverse range of functionality [70]. However, more extreme structural manipulations, such as aggregation, may render a protein nonfunctional. Molecular chaperones and proteolysis machinery function as a proteostasis network (PN) to preserve the proper structure of more than 10,000 proteins in the human body [71]. Key PN components became insoluble in AD, including HSPs (Fig. 5) and multiple members of the UPP: UBB, UBC, USP14, UCHL1, UBA52, and UBA1 (Tables 1, 2, and S7 and Fig. 4). It is likely that reduced/lost functionality of these PN components may be a major contribution to the diverse protein aggregation observed in this study. This, in turn, may create an environment where many proteins are unable to maintain proper structure, leading to reduced/lost functionality for a multitude of cellular processes. Furthermore, an increasing cycle of proteome imbalance may lead to PN collapse and cell death [71]. This possibility is increased by the fact that glycolysis was the most significantly overrepresented gene ontology biological process associated with the alteration of protein aggregation between AD and control patients (Fig. 2), implying bioenergetic deficits.

## Conclusions

Diverse proteins became more detergent-insoluble in the brains of both AD and MCI patients compared to age-matched controls, including those involved in energetics, proteolysis, and DNA damage repair. Additionally, a number of these proteins migrated on SDS-PAGE far above their predicted molecular masses, suggesting formation of stable aggregates. These observations make it quite likely that protein aggregates in AD and MCI pose a direct toxic insult to neurons in addition to reducing the efficiency of other critical aspects of cell physiology. Pharmaceutical interventions that increase autophagy [4–6] may provide a useful therapeutic treatment to combat protein aggregation.

## Supplementary information

**Additional file 1: Figure S1.** AD and control brains have similar amounts of soluble protein. Amido black stained Western blot of control and AD cortex RIPA-soluble (supernatant) fractions. Each lane is a cortical sample from one individual, presented in the same order as the cases in Table S1 (1–16). The ratios reflecting the total protein in the various quadrants are presented along with tubulin. The 51 kDa protein (tubulin) was excluded from the analysis of each quadrant. *P* values are given. Data are presented as mean ± SEM (*n* = 8/group).

**Additional file 2: Figure S2.** The MCI brain has increased amounts of insoluble protein. Amido black stained Western blot of control and MCI cortex RIPA-insoluble fractions (aggregates). Each lane is a cortical sample from one individual, presented in the same order as the cases in Table S2 (17–36). The ratios reflecting the total protein in the various quadrants are presented along with tubulin. The 51 kDa protein (tubulin) was excluded from the analysis of each quadrant. *P* values are given. Data are presented as mean ± SEM (*n* = 10/group).

**Additional file 3: Figure S3.** MCI and control brains have similar amounts of soluble protein. Amido black stained Western blot of control and MCI cortex RIPA-soluble (supernatant) fractions. Each lane is a cortical sample from one individual, presented in the same order as the cases in Table S2 (17–36). The ratios reflecting the total protein in the various quadrants are presented along with tubulin. The 51 kDa protein (tubulin) was excluded from the analysis of each quadrant. *P* values are given. Data are presented as mean ± SEM (*n* = 10/group).

**Additional file 4: Figure S4.** Increased levels of enzymes in AD brain pelletomes. Western blots and quantifications of enzymes identified by proteomics. A) Glucose-6-phosphate isomerase (GPI). B) Creatine kinase B (CKB). C) Low density lipoprotein receptor-related protein (LRP). D) Adenylate cyclase isozyme 1 (ADCY1). E) Calcium/Calmodulin protein kinase 2 (CAMK2). F) Fatty acid synthase (FASN). G) Phosphoglycerate mutase (PGAM). H) Ubiquitin carboxyl-terminal hydrolase isozyme L1 (UCHL1). I) KU70. *P* values are given. Data are presented as mean ± SEM (*n* = 8/group).

**Additional file 5: Figure S5.** Increased levels of enzymes in MCI brain pelletomes. Western blots and quantifications of enzymes identified by proteomics. A) Glucose-6-phosphate isomerase (GPI). B) Creatine kinase B (CKB). C) Low density lipoprotein receptor-related protein (LRP). D) Adenylate cyclase isozyme 1 (ADCY1). E) Calcium/Calmodulin protein kinase 2 (CAMK2). F) Fatty acid synthase (FASN). G) Phosphoglycerate mutase (PGAM). H) Ubiquitin carboxyl-terminal hydrolase isozyme L1 (UCHL1). I) KU70. *P* values are given. Data are presented as mean ± SEM (*n* = 10/group).

**Additional file 6: Figure S6.** Increased levels of heat-shock proteins in AD brain pelletomes. Western blots and quantifications of heat-shock proteins. A) HSP90β. B) HSP75. C) HSP70. D) HSP40. *P* values are given. Data are presented as mean ± SEM (*n* = 8/group).

**Additional file 7: Figure S7.** Increased levels of heat-shock protein 70 in MCI brain pelletomes. Western blots and quantifications of heat-shock proteins. A) HSP90β. B) HSP75. C) HSP70. D) HSP40. *P* values are given. Data are presented as mean ± SEM (*n* = 10/group).

**Additional file 8: Figure S8.** Altered levels of structural proteins in AD brain pelletomes. Western blots and quantifications of structural proteins identified by proteomics. A) Coronin. B) Drebrin. C) Tubulin. D) Gelsolin. E) Dynamin. F) 14–3-3. For the AD pelletome fraction, 14–3-3 is observed at its predicted molecular weight (28 kDa) and at 220 kDa (HMW). *P* values are given. Data are presented as mean ± SEM (*n* = 8/group).

**Additional file 9: Figure S9.** Altered levels of structural proteins in MCI brain pelletomes. Western blots and quantifications of structural proteins identified by proteomics. A) Coronin. B) Drebrin. C) Tubulin. D) Gelsolin. E) Dynamin. F) 14–3-3. *P* values are given. Data are presented as mean ± SEM (*n* = 10/group).

**Additional file 10: Figure S10.** Altered levels of secondary protein modifications in MCI brain pelletomes. Western blots and quantifications of protein secondary modifications. A) Carboxymethyl-lysine (CML). B) Advanced glycation end products (AGE). C) Methylglyoxal (MG). D) 4-hydroxynonenal (HNE). E) Ubiquitin. F) Acetyl-lysine (AcLys). *P* values are given. Data are presented as mean ± SEM (*n* = 10/group).

**Additional file 11: Figure S11.** Aggregation of low molecular weight proteins. Gel with fractions marked for cutting. LC/MS/MS data from fraction 1 is listed in Table S7. The first lane on the left (P8) is AD and the second (P9) is the age- and sex-matched control pelletome. Complete LC/MS/MS data from fractions 1–10 are listed in Table S8.

**Additional file 12: Table S1.** Demographics of AD and control individuals used for proteomics analysis and Western blot studies. Controls were classified as normal individuals.

**Additional file 13: Table S2.** Demographics of MCI and control individuals used for Western blot studies. Controls were classified as normal individuals.

**Additional file 14: Table S3.** All proteins ranked by spectral counts (using method from Table 1).

**Additional file 15: Table S4.** All proteins normalized to soluble fraction and then ranked by spectral counts (using method from Table 2).

**Additional file 16: Table S5.** Correlation Analysis between the disease-associated proteins and pelletome proteins enriched in MCI relative to control patients.

**Additional file 17: Table S6.** Correlation Analysis between the disease-associated proteins and pelletome proteins enriched in AD relative to control patients.

**Additional file 18: Table S7.** Lower molecular weight proteins that migrate above 200 kDa on SDS-polyacrylamide gels.

**Additional file 19: Table S8.** Proteins in each fraction of cut gel ranked by spectral counts. Gel is shown in Figure S11.

## Data Availability

All data generated during this study are included in this published article and its supplementary information files.

## Abbreviations

Aβ: Amyloid beta
AD: Alzheimer’s disease
ADCY1: Adenylate cyclase isoenzyme 1
AGEs: Advanced glycation end products
ApoE: Apolipoprotein E
APP: Amyloid precursor protein
CAMK2: Calcium/calmodulin-dependent protein kinase 2 gamma
CKB: Creatine kinase B
CML: Carboxymethyl-lysine
DTT: Dithiothreitol
FASN: Fatty acid synthase
GO: Gene ontology
GPI: Glucose-6-phosphate isomerase
HNE: 4-Hydroxynonenal
HSP: Heat shock protein
LC/MS/MS: Liquid chromatography-tandem mass spectrometry
LRP: Low density lipoprotein receptor-related protein
MAP2K1: Mitogen-activated protein kinase kinase 1
MCI: Mild cognitively impaired
MG: Methylglyoxal
MMSE: Mini-mental state examination
mw: Molecular weight
NFTs: Neurofibrillary tangles
PBS: Phosphate buffered saline
PCA: Principle component analysis
PGAM: Phosphoglycerate mutase 1
PM: Postmortem
PN: Proteostasis network
RAP1GDS1: Rap1 GTPase-GDP dissociation stimulator 1
RIPA: Radioimmunoprecipitation assay buffer
SDS-PAGE: Sodium dodecyl sulfate-polyacrylamide gel electrophoresis
UBA1: Ubiquitin-like modifier activating enzyme 1
UBA52: Ubiquitin-60S ribosomal protein L40
UBB: Ubiquitin
UBC: Polyubiquitin-C
UCHL1: Ubiquitin carboxyl-terminal hydrolase isoenzyme L1
UPP: Ubiquitin proteasome pathway
USP14: Ubiquitin carboxyl-terminal hydrolase 14
VPS35: Vacuolar protein sorting-associated protein 35
