# Supplementary figures and images for: Diverse proteins aggregate in mild cognitive impairment and Alzheimer’s disease brain
Source: Alzheimers Res Ther. 2020 Jun 19;12:75. doi: 10.1186/s13195-020-00641-2 (PMC7305608; doi:10.1186/s13195-020-00641-2)

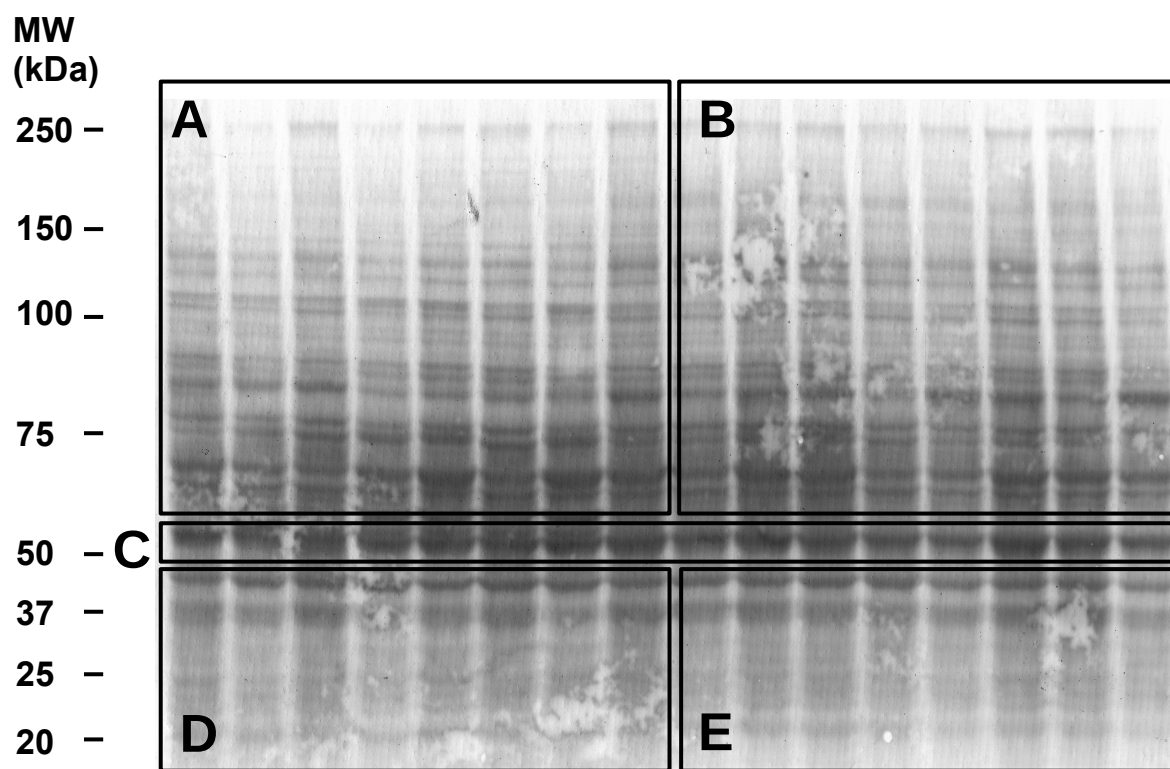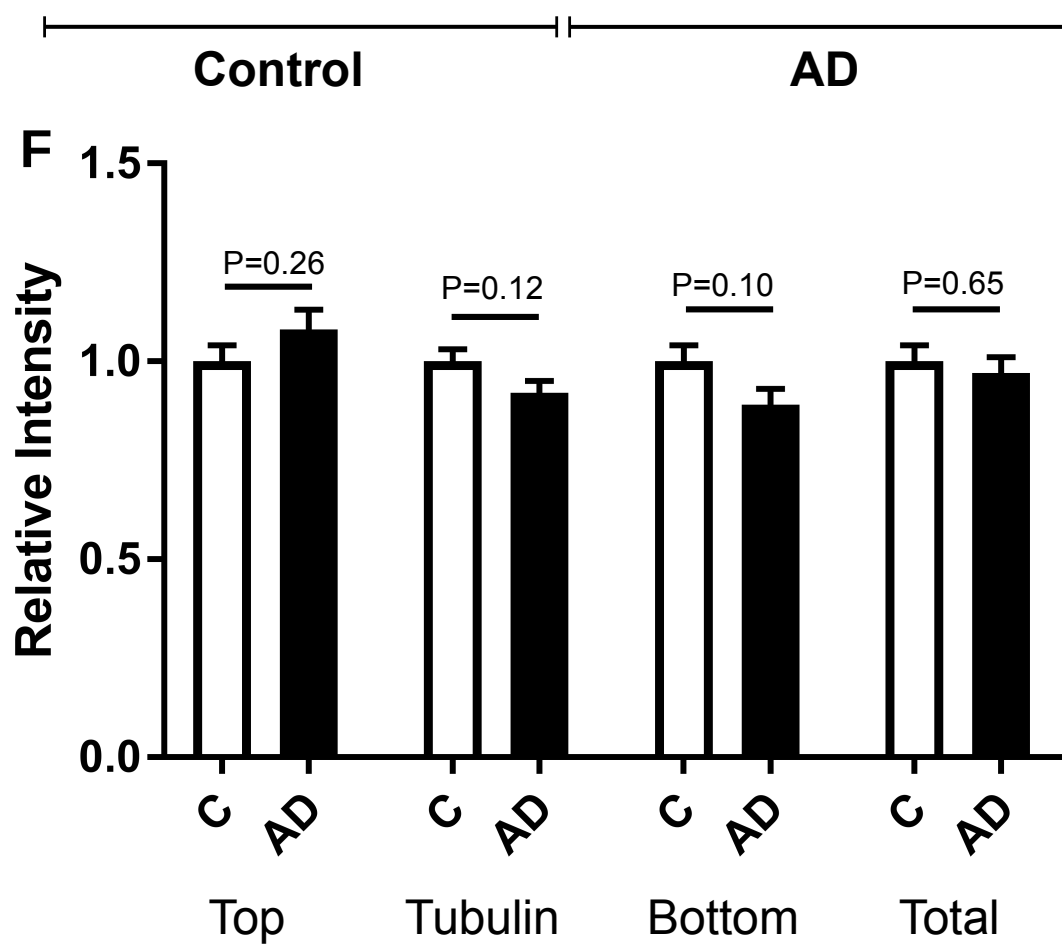

Supplement: Supplementary file 1 — Additional file 1: Figure S1. AD and control brains have similar amounts of soluble protein. Amido black stained Western blot of control and AD cortex RIPA-soluble (supernatant) fractions. Each lane is a cortical sample from one individual, presented in the same order as the cases in Table S1 (1–16). The ratios reflecting the total protein in the various quadrants are presented along with tubulin. The 51 kDa protein (tubulin) was excluded from the analysis of each quadrant. P values are given. Data are presented as mean ± SEM (n = 8/group). [file 13195_2020_641_MOESM1_ESM.pdf]

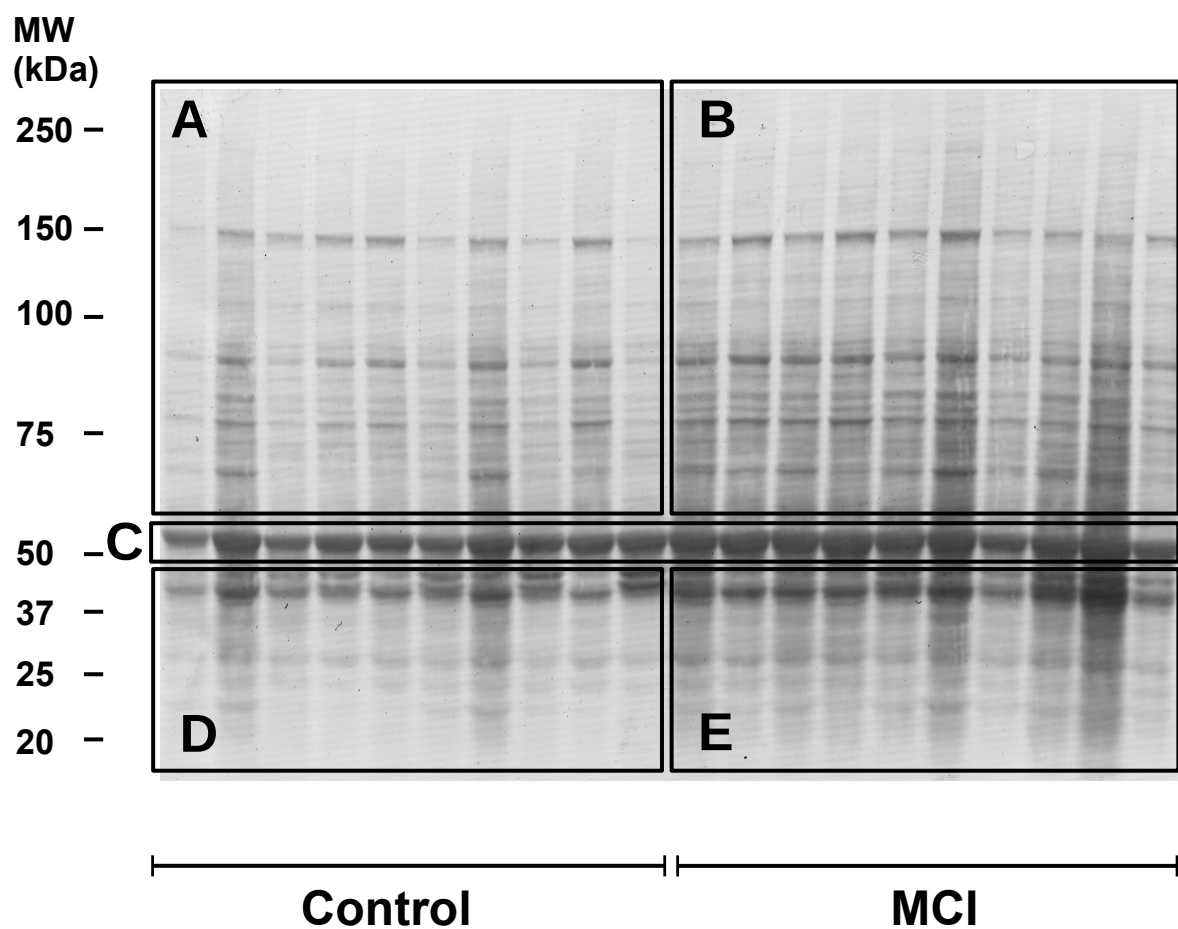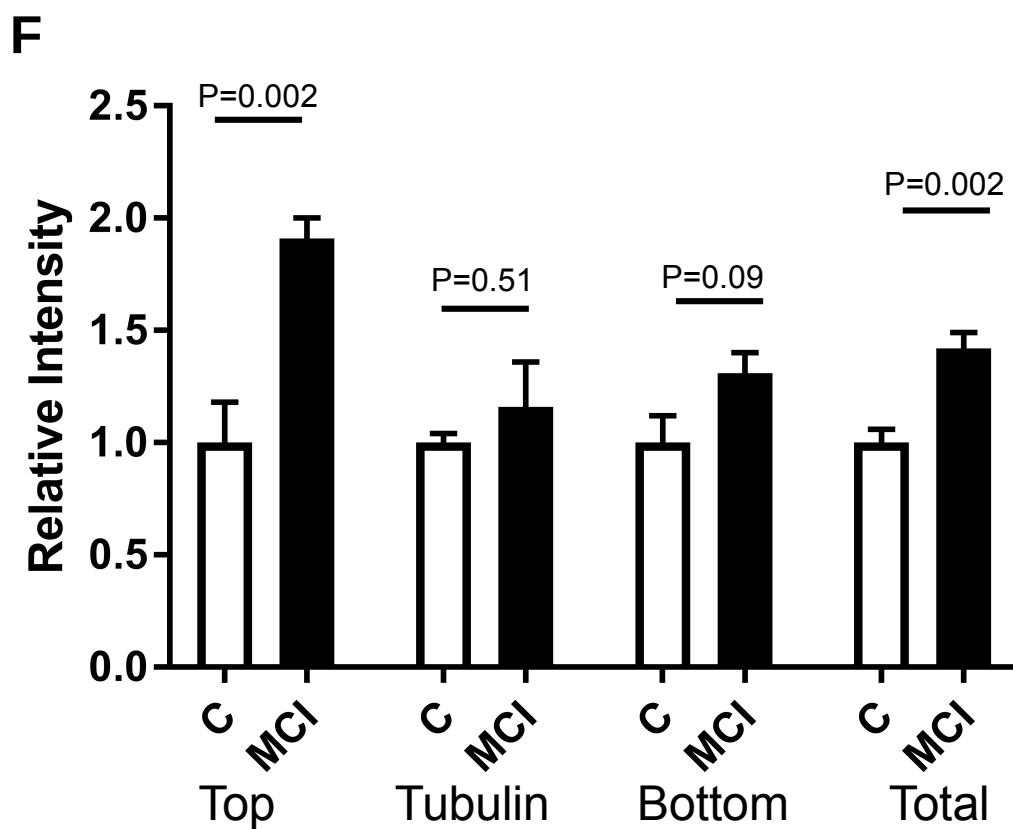

Supplement: Supplementary file 2 — Additional file 2: Figure S2. The MCI brain has increased amounts of insoluble protein. Amido black stained Western blot of control and MCI cortex RIPA-insoluble fractions (aggregates). Each lane is a cortical sample from one individual, presented in the same order as the cases in Table S2 (17–36). The ratios reflecting the total protein in the various quadrants are presented along with tubulin. The 51 kDa protein (tubulin) was excluded from the analysis of each quadrant. P values are given. Data are presented as mean ± SEM (n = 10/group). [file 13195_2020_641_MOESM2_ESM.pdf]

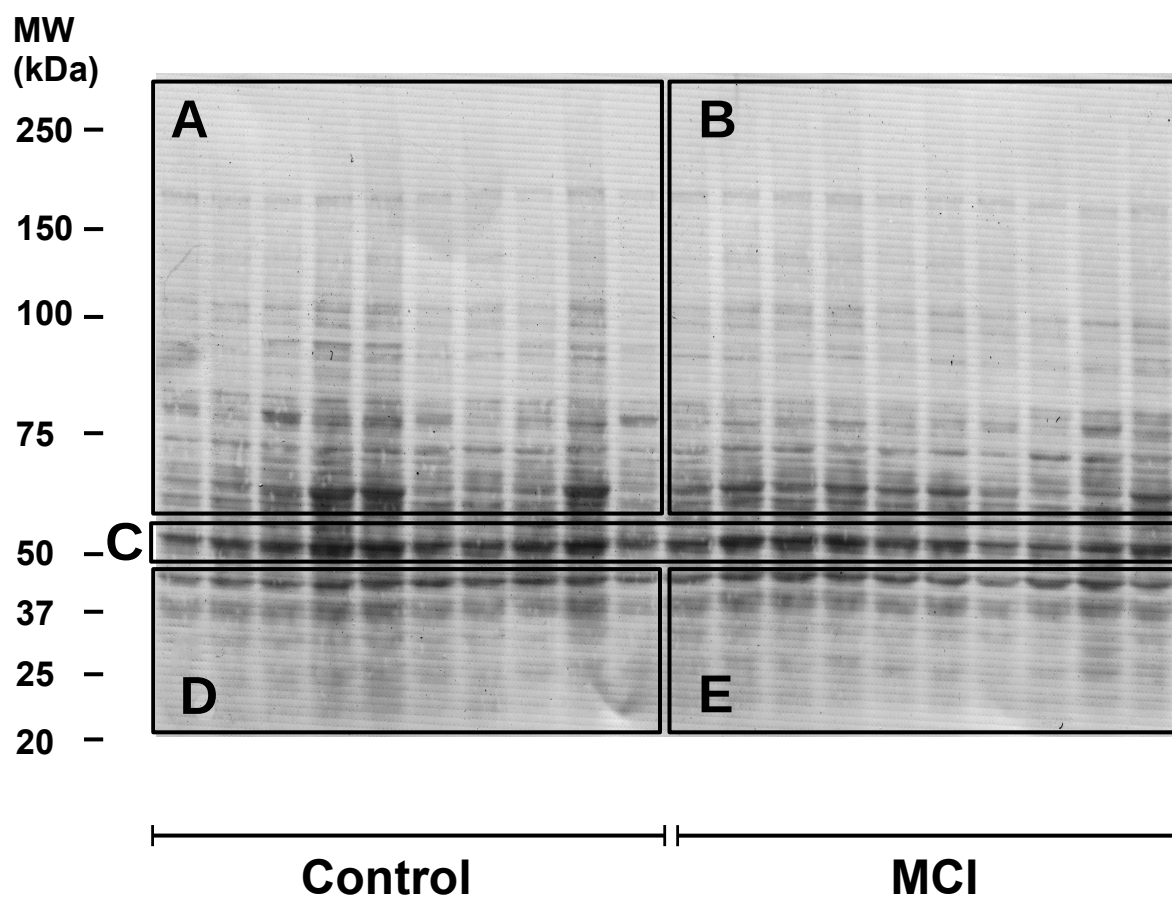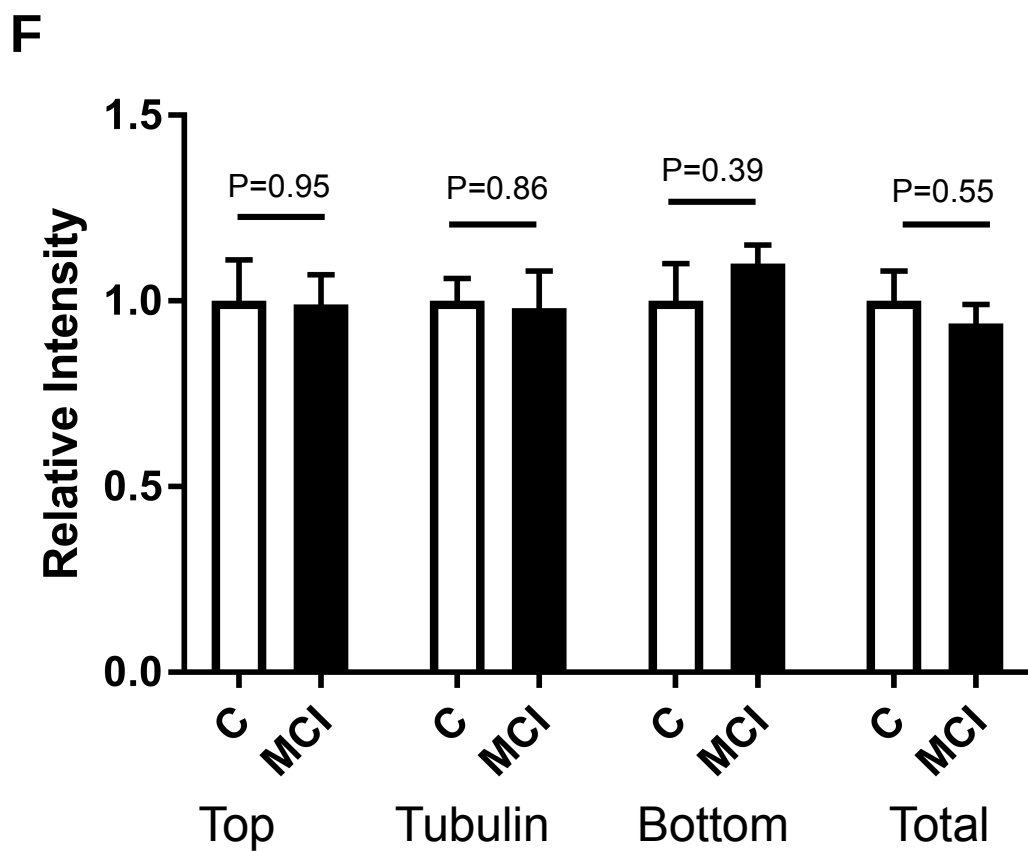

Supplement: Supplementary file 3 — Additional file 3: Figure S3. MCI and control brains have similar amounts of soluble protein. Amido black stained Western blot of control and MCI cortex RIPA-soluble (supernatant) fractions. Each lane is a cortical sample from one individual, presented in the same order as the cases in Table S2 (17–36). The ratios reflecting the total protein in the various quadrants are presented along with tubulin. The 51 kDa protein (tubulin) was excluded from the analysis of each quadrant. P values are given. Data are presented as mean ± SEM (n = 10/group). [file 13195_2020_641_MOESM3_ESM.pdf]

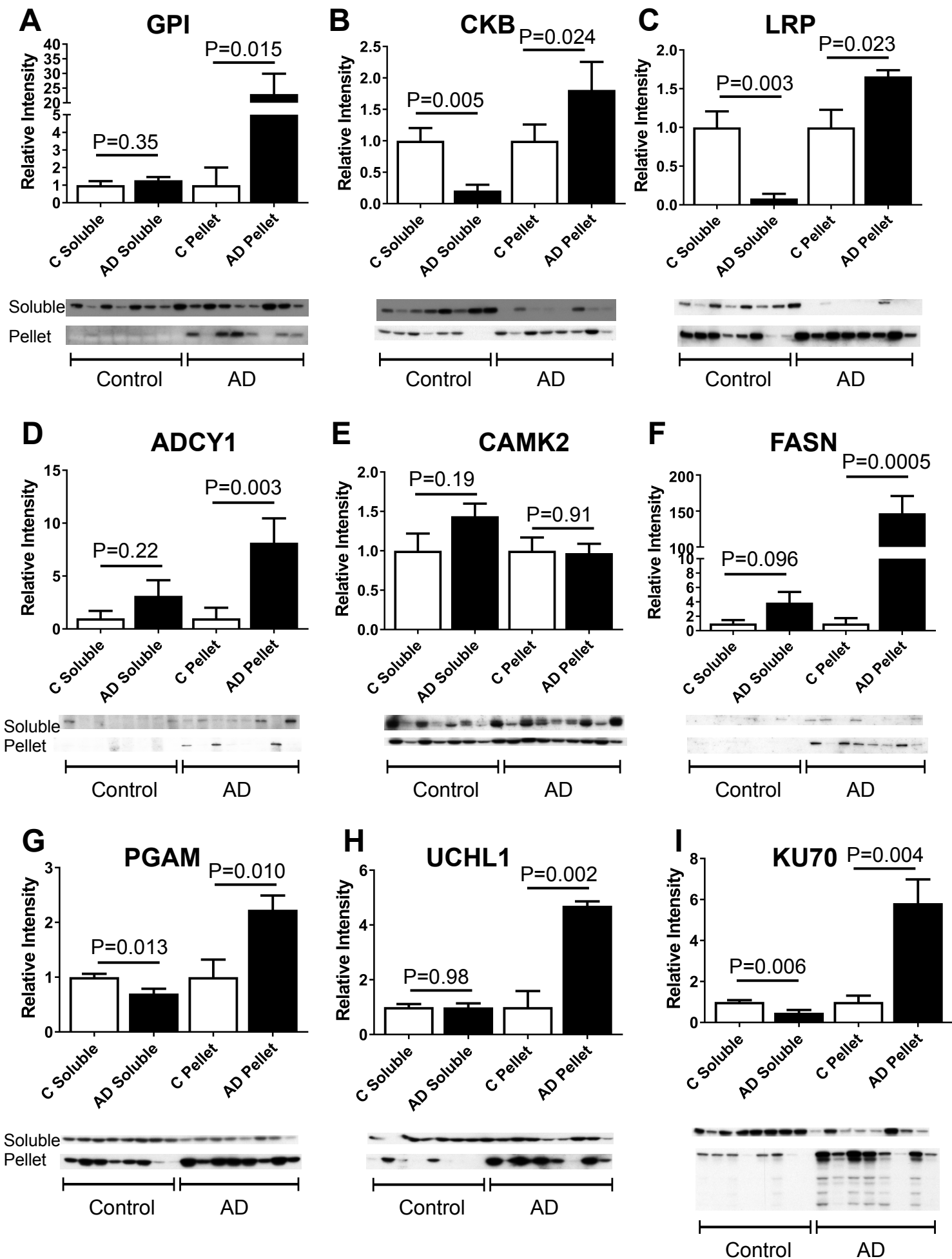

Supplement: Supplementary file 4 — Additional file 4: Figure S4. Increased levels of enzymes in AD brain pelletomes. Western blots and quantifications of enzymes identified by proteomics. A) Glucose-6-phosphate isomerase (GPI). B) Creatine kinase B (CKB). C) Low density lipoprotein receptor-related protein (LRP). D) Adenylate cyclase isozyme 1 (ADCY1). E) Calcium/Calmodulin protein kinase 2 (CAMK2). F) Fatty acid synthase (FASN). G) Phosphoglycerate mutase (PGAM). H) Ubiquitin carboxyl-terminal hydrolase isozyme L1 (UCHL1). I) KU70. P values are given. Data are presented as mean ± SEM (n = 8/group). [file 13195_2020_641_MOESM4_ESM.pdf]

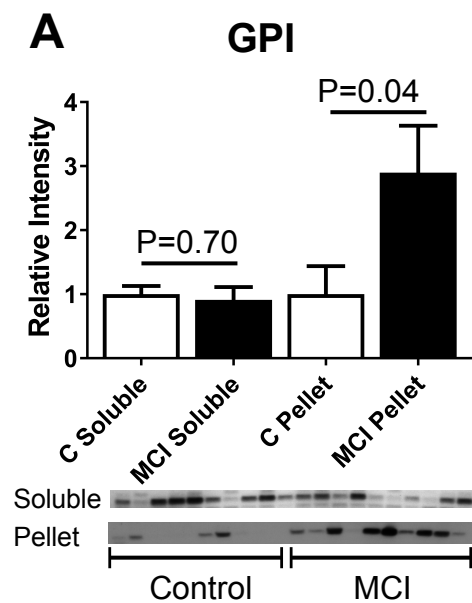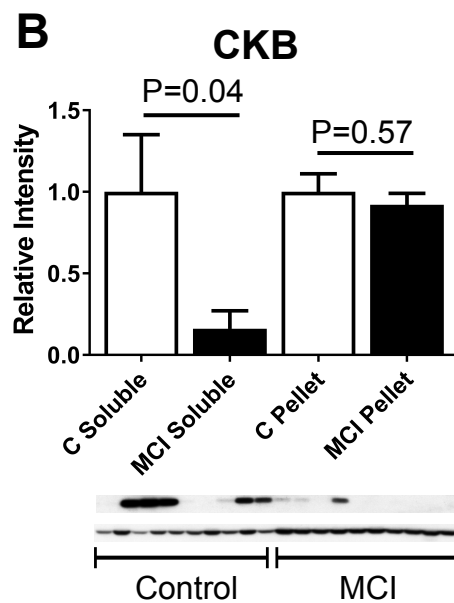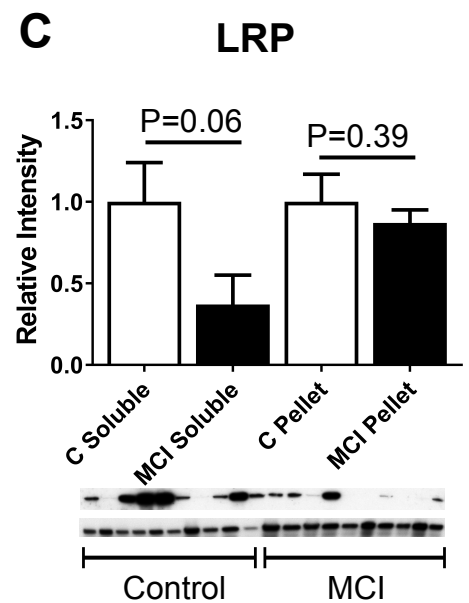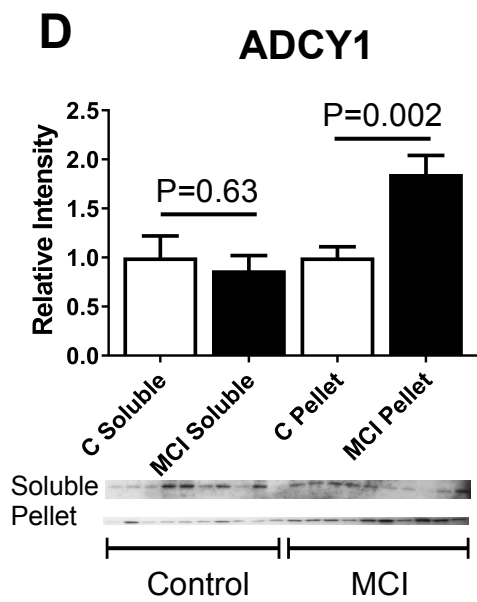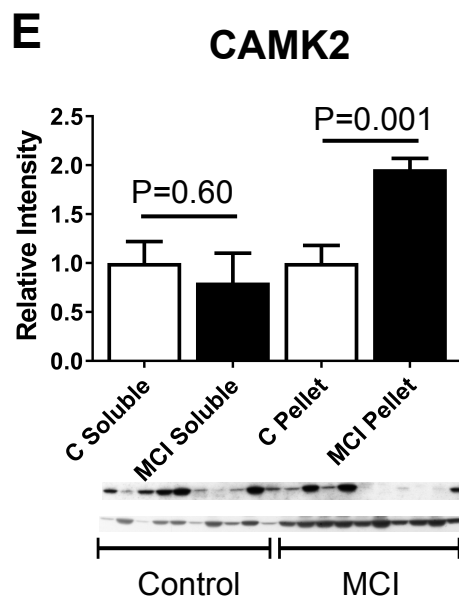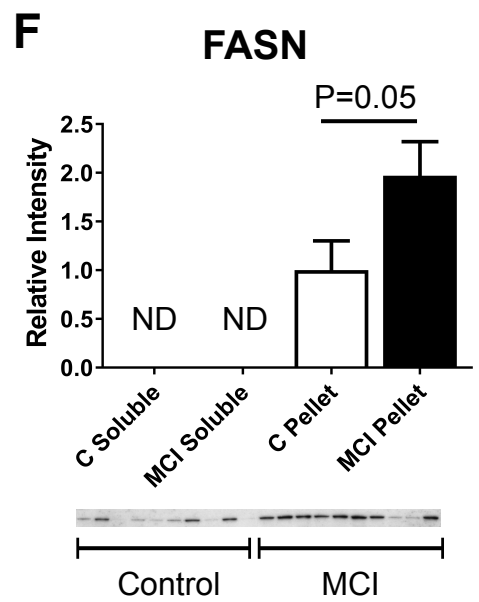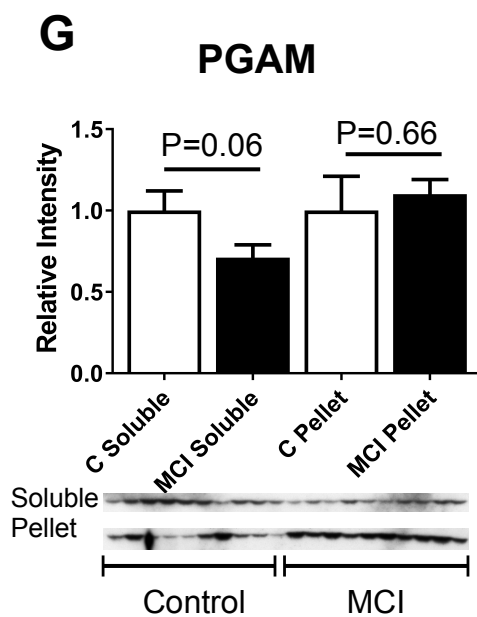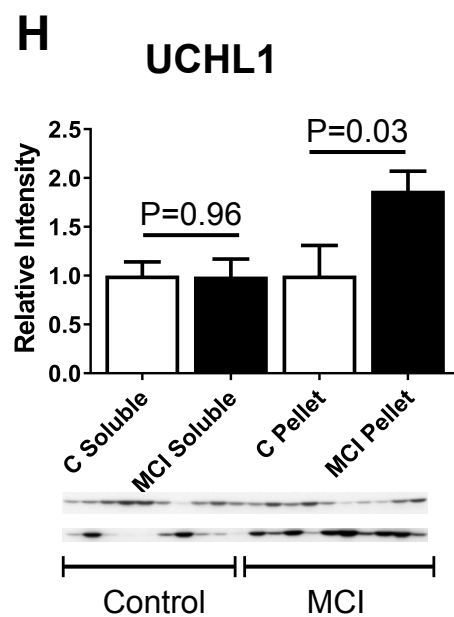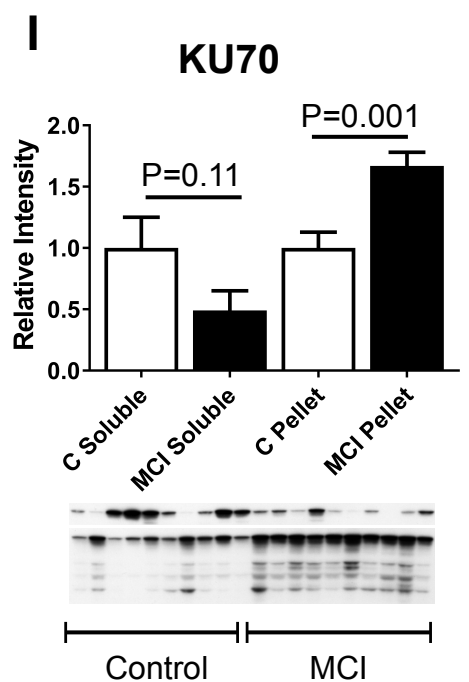

Supplement: Supplementary file 5 — Additional file 5: Figure S5. Increased levels of enzymes in MCI brain pelletomes. Western blots and quantifications of enzymes identified by proteomics. A) Glucose-6-phosphate isomerase (GPI). B) Creatine kinase B (CKB). C) Low density lipoprotein receptor-related protein (LRP). D) Adenylate cyclase isozyme 1 (ADCY1). E) Calcium/Calmodulin protein kinase 2 (CAMK2). F) Fatty acid synthase (FASN). G) Phosphoglycerate mutase (PGAM). H) Ubiquitin carboxyl-terminal hydrolase isozyme L1 (UCHL1). I) KU70. P values are given. Data are presented as mean ± SEM (n = 10/group). [file 13195_2020_641_MOESM5_ESM.pdf]

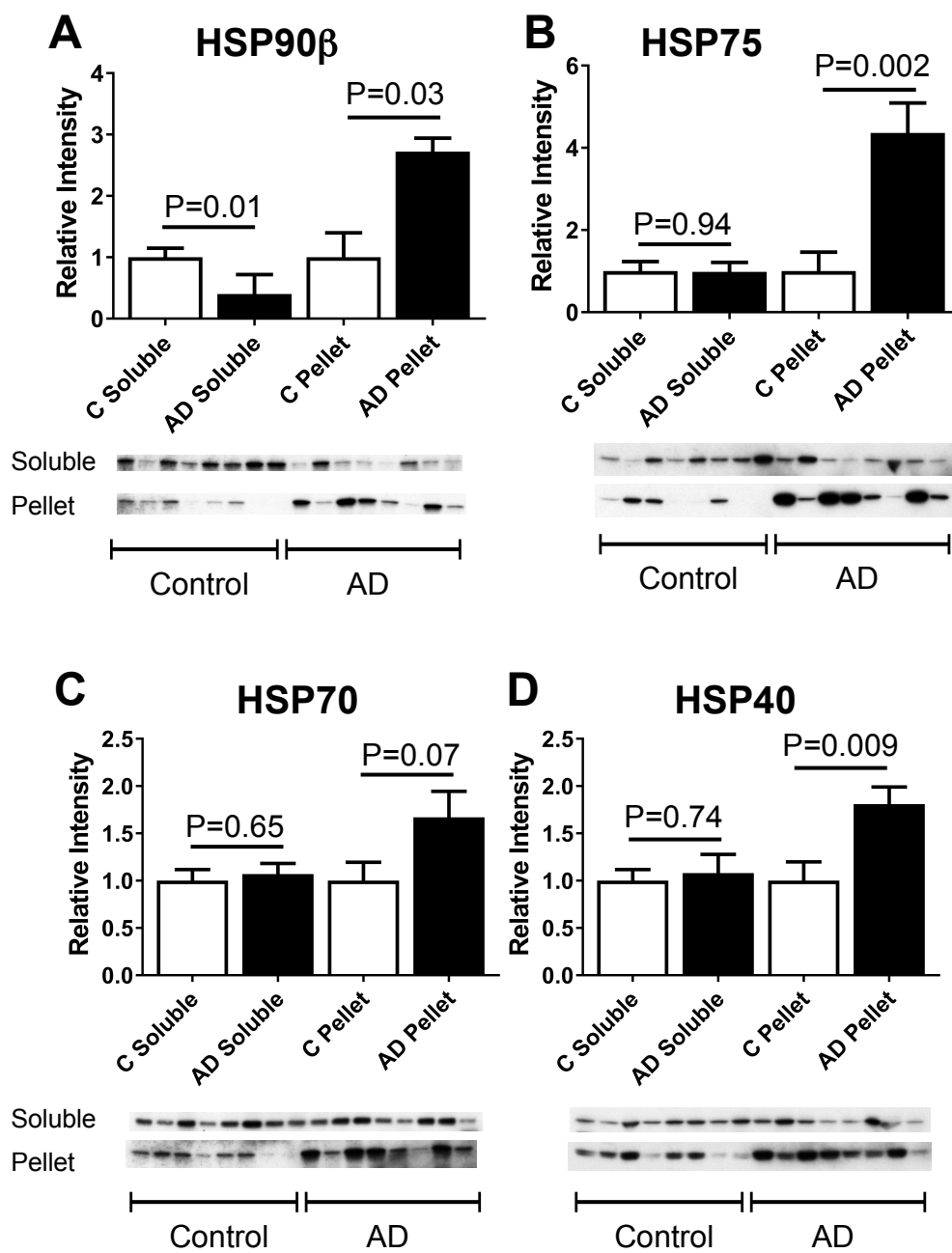

Supplement: Supplementary file 6 — Additional file 6: Figure S6. Increased levels of heat-shock proteins in AD brain pelletomes. Western blots and quantifications of heat-shock proteins. A) HSP90β. B) HSP75. C) HSP70. D) HSP40. P values are given. Data are presented as mean ± SEM (n = 8/group). [file 13195_2020_641_MOESM6_ESM.pdf]

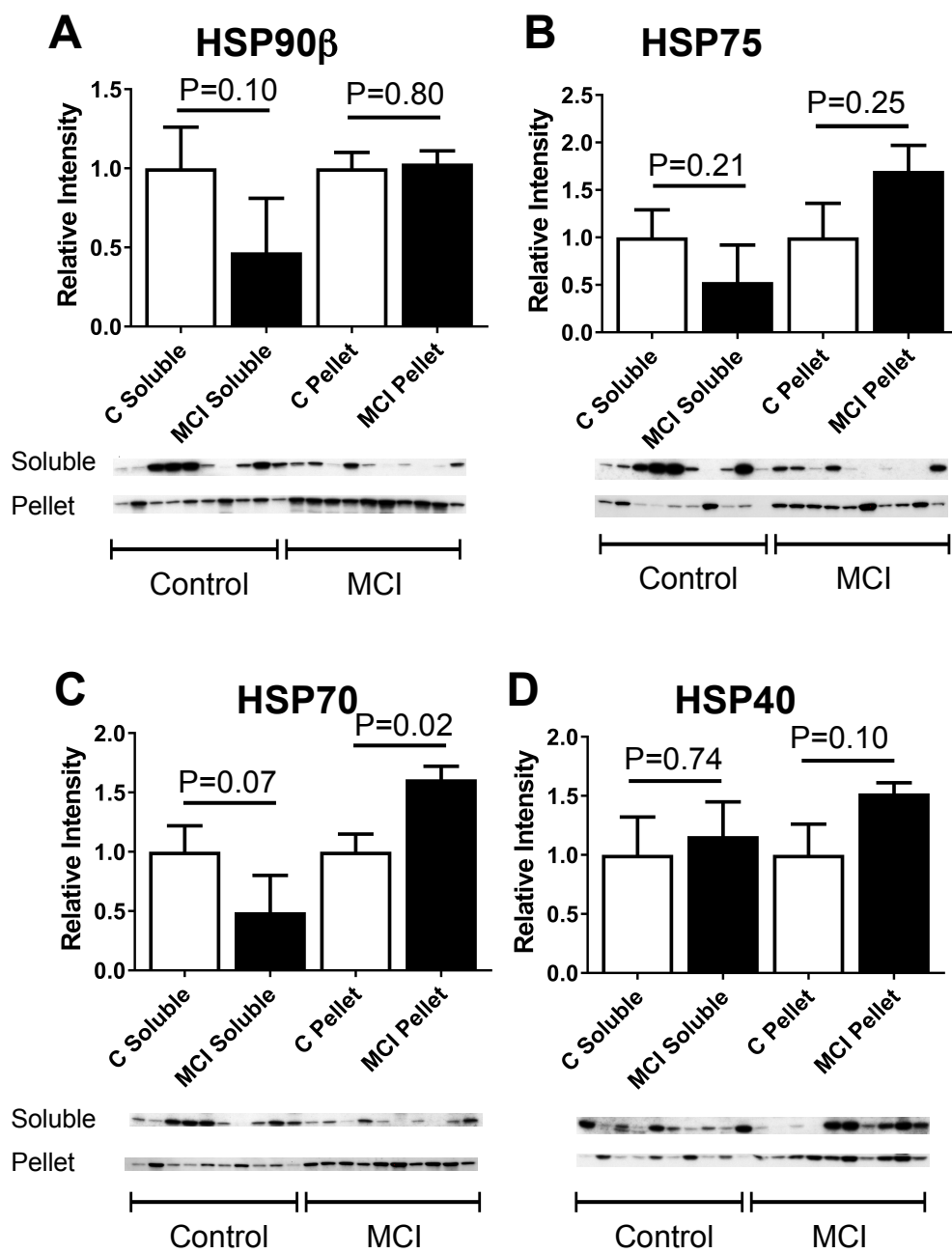

Supplement: Supplementary file 7 — Additional file 7: Figure S7. Increased levels of heat-shock protein 70 in MCI brain pelletomes. Western blots and quantifications of heat-shock proteins. A) HSP90β. B) HSP75. C) HSP70. D) HSP40. P values are given. Data are presented as mean ± SEM (n = 10/group). [file 13195_2020_641_MOESM7_ESM.pdf]

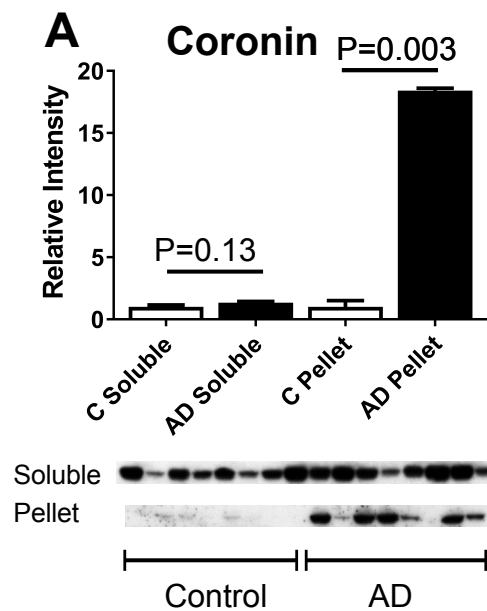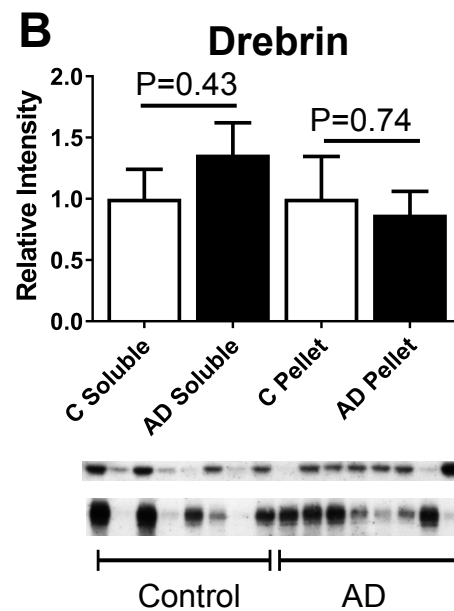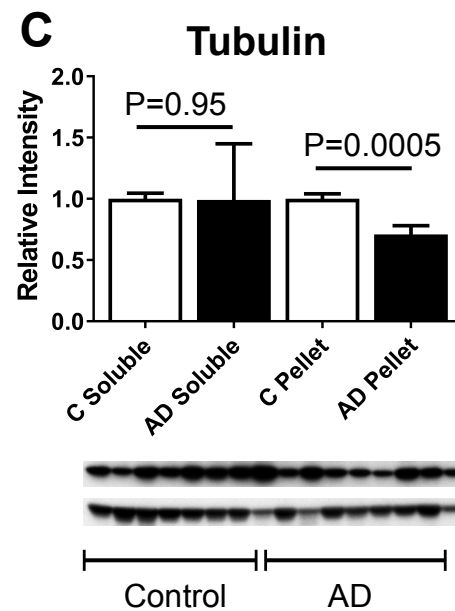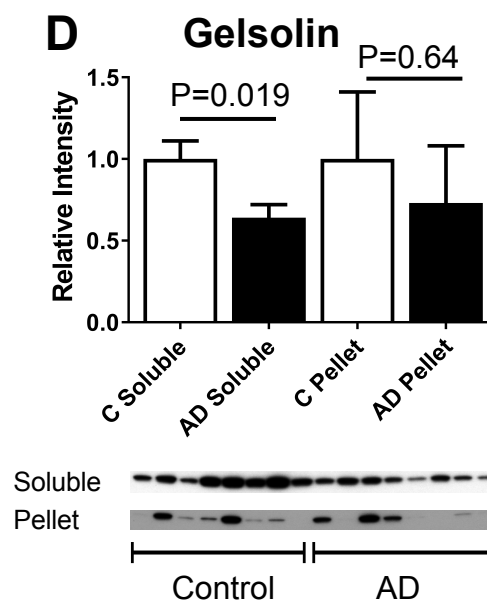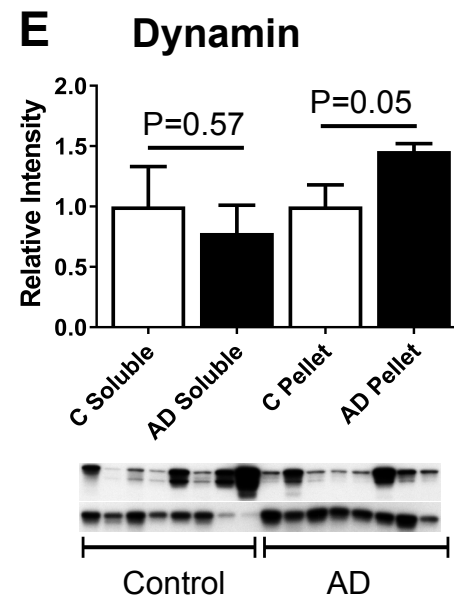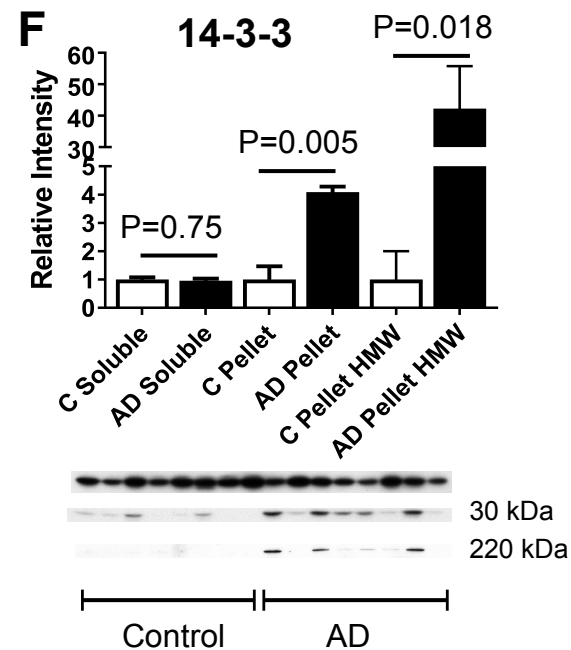

Supplement: Supplementary file 8 — Additional file 8: Figure S8. Altered levels of structural proteins in AD brain pelletomes. Western blots and quantifications of structural proteins identified by proteomics. A) Coronin. B) Drebrin. C) Tubulin. D) Gelsolin. E) Dynamin. F) 14–3-3. For the AD pelletome fraction, 14–3-3 is observed at its predicted molecular weight (28 kDa) and at 220 kDa (HMW). P values are given. Data are presented as mean ± SEM (n = 8/group). [file 13195_2020_641_MOESM8_ESM.pdf]

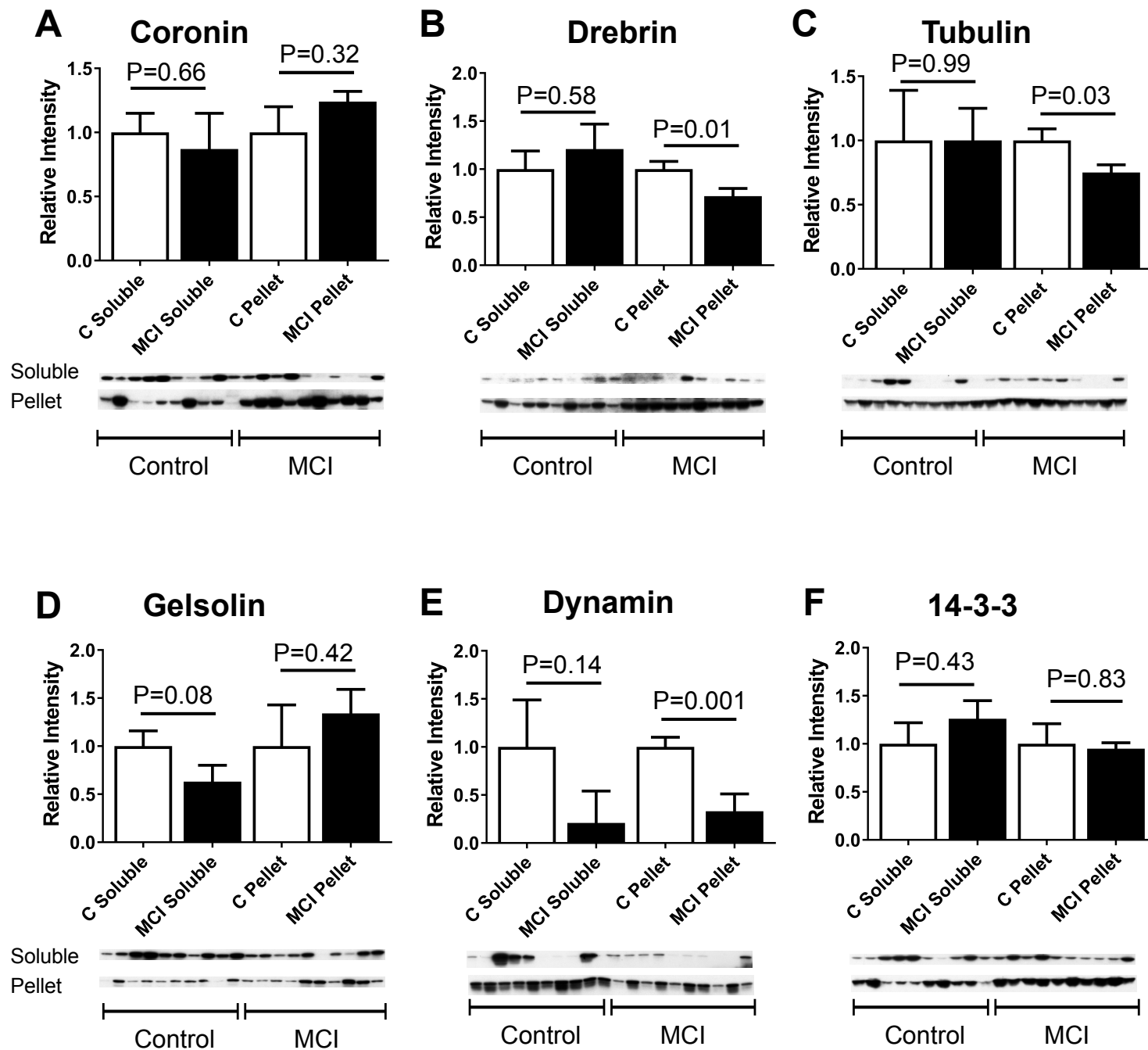

Supplement: Supplementary file 9 — Additional file 9: Figure S9. Altered levels of structural proteins in MCI brain pelletomes. Western blots and quantifications of structural proteins identified by proteomics. A) Coronin. B) Drebrin. C) Tubulin. D) Gelsolin. E) Dynamin. F) 14–3-3. P values are given. Data are presented as mean ± SEM (n = 10/group). [file 13195_2020_641_MOESM9_ESM.pdf]

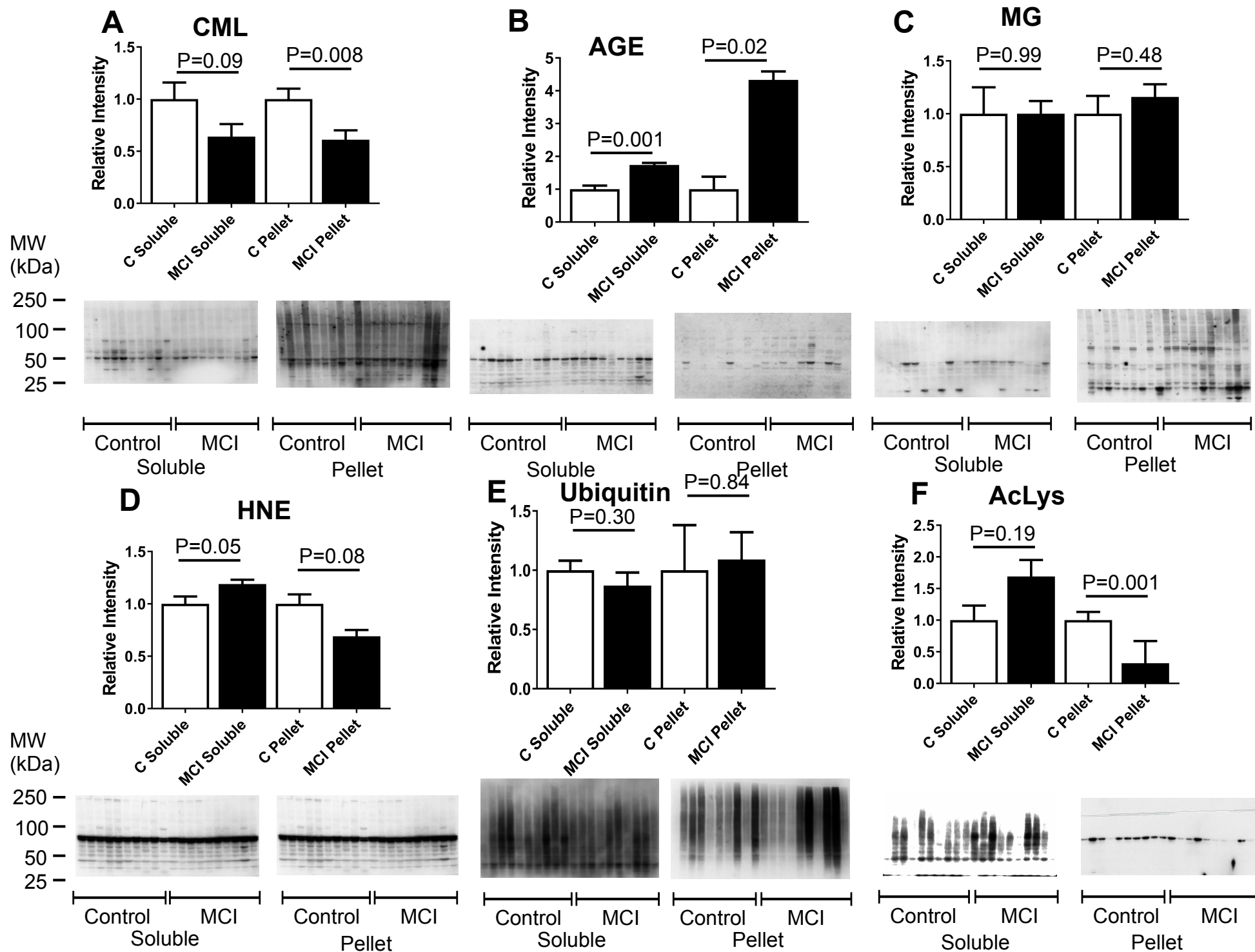

Supplement: Supplementary file 10 — Additional file 10: Figure S10. Altered levels of secondary protein modifications in MCI brain pelletomes. Western blots and quantifications of protein secondary modifications. A) Carboxymethyl-lysine (CML). B) Advanced glycation end products (AGE). C) Methylglyoxal (MG). D) 4-hydroxynonenal (HNE). E) Ubiquitin. F) Acetyl-lysine (AcLys). P values are given. Data are presented as mean ± SEM (n = 10/group). [file 13195_2020_641_MOESM10_ESM.pdf]

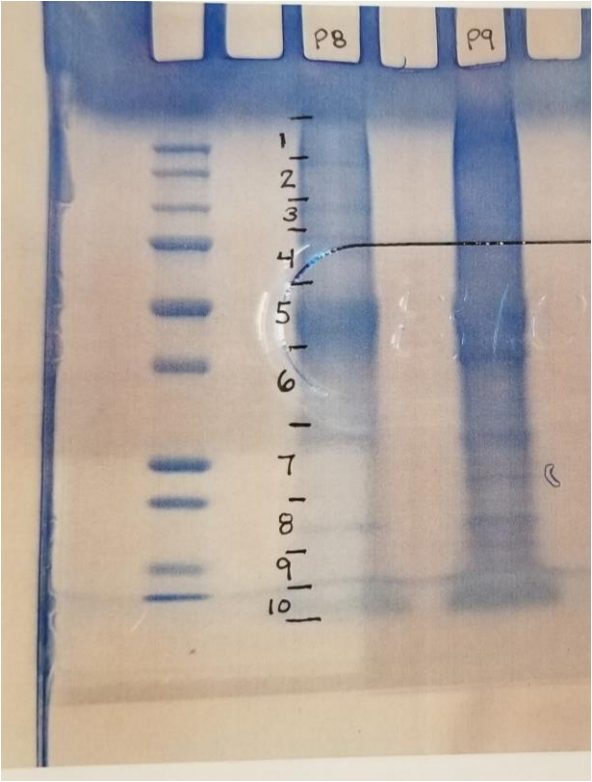

Supplement: Supplementary file 11 — Additional file 11: Figure S11. Aggregation of low molecular weight proteins. Gel with fractions marked for cutting. LC/MS/MS data from fraction 1 is listed in Table S7. The first lane on the left (P8) is AD and the second (P9) is the age- and sex-matched control pelletome. Complete LC/MS/MS data from fractions 1–10 are listed in Table S8. [file 13195_2020_641_MOESM11_ESM.pdf]
