# Supplementary material for: Diverse proteins aggregate in mild cognitive impairment and Alzheimer’s disease brain
Source: Alzheimers Res Ther. 2020 Jun 19;12:75. doi: 10.1186/s13195-020-00641-2 (PMC7305608; doi:10.1186/s13195-020-00641-2)
Supplement: Supplementary file 12 — Additional file 12: Table S1. Demographics of AD and control individuals used for proteomics analysis and Western blot studies. Controls were classified as normal individuals. [file 13195_2020_641_MOESM12_ESM.docx]

| **Case Number** | **Diagnosis** | **Age** | **Sex** | **PM (hrs)** | **ApoE** | **Braak1** | **Blessed** | **MMSE** |
| --- | --- | --- | --- | --- | --- | --- | --- | --- |
| 1x5070 | Control | 97 | F | 12 | 3/3 | 1 | 2 | 26 |
| 2x5302 | Control | 83 | F | 72 | 2/4 | 1 |  | 29 |
| 3x5512 | Control | 89 | F | 12 | 2/3 | 2 |  | 30 |
| 4x5049 | Control | 102 | F | 9 | 3/3 | 1 |  | 27 |
| 5x4870 | Control | 63 | F | 8 | 3/3 | 1 | 0 | 30 |
| 6x4689 | Control | 79 | F | 6 | 3/3 | 0 | 1 | 27 |
| 7x5248 | Control | 93 | F | 18 | 3/3 | 1 |  | 30 |
| 8x5447 | Control | 91 | F | 8 | 3/3 | 1 |  | 28 |
| 9x5759 | AD | 87 | F | 18 | 3/3 | 6 | 9 | 18 |
| 10x5798 | AD | 87 | F | 7 | 3/3 | 5 | 3 | 11 |
| 11x5799 | AD | 82 | F | 15 |  | 6 | 26 | 9 |
| 12x5764 | AD | 82 | F | 8 | 3/4 | 6 | 33 | 23 |
| 13x5763 | AD | 94 | F | 24 | 3/4 | 5 | 25 | 13 |
| 14x5792 | AD | 93 | F | 10 | 3/3 | 5 | 8 | 22 |
| 15x5761 | AD | 89 | F |  | 3/4 | 5 | 3 | 25 |
| 16x5755 | AD | 83 | F | 12 | 4/4 | 6 | 18 | 19 |

**Table S1:** Demographics of AD and control individuals used for proteomics analysis and Western blot studies. Controls were classified as normal individuals.
