# Supplementary material for: Diverse proteins aggregate in mild cognitive impairment and Alzheimer’s disease brain
Source: Alzheimers Res Ther. 2020 Jun 19;12:75. doi: 10.1186/s13195-020-00641-2 (PMC7305608; doi:10.1186/s13195-020-00641-2)
Supplement: Supplementary file 13 — Additional file 13: Table S2. Demographics of MCI and control individuals used for Western blot studies. Controls were classified as normal individuals. [file 13195_2020_641_MOESM13_ESM.docx]

| **Case Number** | **Diagnosis** | **Age** | **Sex** | **PM (hrs)** | **ApoE** | **Braak1** | **Blessed** | **MMSE** |
| --- | --- | --- | --- | --- | --- | --- | --- | --- |
| 17x5398 | Control | 89 | F | 14 | 2/3 | 0 |  | 30 |
| 18x5341 | Control | 77 | F | 12 |  | 0 |  |  |
| 19x5318 | Control |  | F |  | 3/3 | 0 |  |  |
| 20x5281 | Control | 84 | M |  | 3/4 | 0 | 3 | 30 |
| 21x5200 | Control | 76 | F |  |  | 0 |  | 30 |
| 22x5106 | Control |  | F |  |  | 0 |  |  |
| 23x5105 | Control | 74 | F | 8 |  | 0 |  |  |
| 24x5006 | Control | 69 | M | 24 |  | 0 | 12 | 21 |
| 25x4954 | Control | 76 | M |  | 3/3 | 0 | 2 | 29 |
| 26x4942 | Control | 83 | M |  | 3/3 | 0 | 3 | 30 |
| 27x5558 | MCI | 89 | F | 18 | 3/3 | 3 | 14 | 17 |
| 28x5195 | MCI | 78 | F |  | 3/4 | 3 | 26 | 8 |
| 29x5046 | MCI | 75 | F | 16 | 3/3 | 2 | 33 | 0 |
| 30x5329 | MCI | 84 | M | 12 | 3/4 | 2 |  |  |
| 31x4903 | MCI | 75 | F |  | 3/4 | 3 | 33 | 0 |
| 32x5187 | MCI | 83 | F | 4 |  | 3 | 10 | 14 |
| 33x5612 | MCI | 72 | F |  | 3/4 | 2 | 8 | 22 |
| 34x4761 | MCI | 69 | M | 48 | 3/4 | 2 | 17 | 13 |
| 35x4662 | MCI | 77 | M |  | 3/3 | 2 | 25 | 11 |
| 36x5395 | MCI | 82 | M | 15 |  | 3 |  | 6 |

**Table S2:** Demographics of MCI and control individuals used for Western blot studies.

Controls were classified as normal individuals.
