# Supplementary material for: Diverse proteins aggregate in mild cognitive impairment and Alzheimer’s disease brain
Source: Alzheimers Res Ther. 2020 Jun 19;12:75. doi: 10.1186/s13195-020-00641-2 (PMC7305608; doi:10.1186/s13195-020-00641-2)
Supplement: Supplementary file 18 — Additional file 18: Table S7. Lower molecular weight proteins that migrate above 200 kDa on SDS-polyacrylamide gels. [file 13195_2020_641_MOESM18_ESM.docx]

| **Protein Name** | **MW** | **AD/C** | **Description** |
| --- | --- | --- | --- |
|  |  |  |  |
| PDXK | 35102 | >50 | Pyridoxal kinase |
| MAP2K1 | 43439 | >50 | Dual specificity mitogen-activated protein kinase kinase 1 |
| ADCY1 | 21635 | >50 | Adenylate cyclase isoenzyme 1 |
| PRKAR2B | 46302 | >50 | cAMP-dependent protein kinase type II-beta regulatory subunit |
| DBNL | 48207 | >50 | Drebrin |
| UBB | 24824 | >50 | Ubiquitin |
| UCHL1 | 24824 | >50 | Ubiquitin carboxyl-terminal hydrolase isozyme L1 |
| TBCB | 27326 | >50 | Tubulin-folding cofactor B |
| PCBP2 | 38580 | >50 | Poly(rC)-binding protein 2 |
| KIAA0513 | 46639 | >50 | Uncharacterized protein KIAA0513 |
| FLOT1 | 47355 | >50 | Flotillin-1 |
| 14-3-3 | 28082 | 6.5 | 14-3-3 protein beta/alpha |
| HSP90AA2 | 39365 | 3.5 | Heat shock protein HSP 90-alpha A2 |
| MAP2K2 | 44424 | 3.5 | Dual specificity mitogen-activated protein kinase kinase 2 |
| SLC25A5 | 32852 | 3.0 | ADP/ATP translocase 2 |
| HSP90β | 44349 | 3.0 | Putative heat shock protein HSP 90-beta 2 |
| CBR1 | 30375 | 2.4 | Carbonyl reductase [NADPH] 1 |
| CNP | 47579 | 1.7 | cyclic-nucleotide-phosphodiesterase |
| PCBP1 | 37498 | 1.5 | Poly(rC)-binding protein 1 |
| PIP4K2A | 46225 | 1.5 | Phosphatidylinositol 5-phosphate 4-kinase type-2 alpha |
| GNAO1 | 40051 | 1.4 | Guanine nucleotide-binding protein G(o) subunit alpha |
| STX1B | 33245 | 1.4 | Syntaxin-1B |
| TUFM | 49542 | 1.3 | Elongation factor Tu, mitochondrial |
| TUBULIN | 49586 | 1.3 | Tubulin beta |
| SLC25A6 | 32866 | 1.2 | ADP/ATP translocase 3 |
| PURA | 34911 | 1.2 | Transcriptional activator protein Pur-alpha |
| CKB | 42644 | 1.2 | Creatine kinase B-type |
| VDAC1 | 30773 | 1.2 | Voltage-dependent anion-selective channel protein |
| GNAZ | 40924 | 1.1 | Guanine nucleotide-binding protein G(z) subunit alpha |
| AP2M1 | 49655 | 1.1 | AP-2 complex subunit mu |
| VDAC2 | 31566 | 1.0 | Voltage-dependent anion-selective channel protein 2 |
| HNRNPA2B1 | 37430 | 1.0 | Heterogeneous nuclear ribonucleoproteins A2/B1 |
| ACTB | 41737 | 1.0 | Actin, cytoplasmic |
| FTL | 20020 | 0.9 | Ferritin light chain |
| HNRNPH1 | 49229 | 0.9 | Heterogeneous nuclear ribonucleoprotein H |
| SYT1 | 47573 | 0.8 | Synaptotagmin-1 |
| MBP | 33117 | 0.8 | Myelin basic protein |
| ACTG2 | 41877 | 0.7 | Actin, gamma |
| GFAP | 49880 | 0.6 | Glial fibrillary acidic protein |
| ACTC1 | 42019 | 0.6 | Actin, alpha |
| PI4KAP1 | 29179 | 0.4 | Putative inactive phosphatidylinositol 4-kinase alpha |

**TABLE S7**: Lower molecular weight proteins that migrate above 200 kDa on SDS-PAGE. Proteins from AD and control cortex were run side by side on SDS-PAGE. The gel was stained with Coomassie blue and each of the two lanes were cut into 10 fractions at the identical levels (Fig. S11). The fractions were analyzed by LC/MS/MS and the spectral counts for each protein were determined. The data show the ratio of AD to control for proteins with a predicted molecular mass less than 51 kDa that migrate above 200 kDa.
